# Phytochemical Investigation and Anti-Inflammatory Activity of the Leaves of *Machilus japonica* var. *kusanoi*

**DOI:** 10.3390/molecules25184149

**Published:** 2020-09-10

**Authors:** Shiou-Ling Li, Ho-Cheng Wu, Tsong-Long Hwang, Chu-Hung Lin, Shuen-Shin Yang, Hsun-Shuo Chang

**Affiliations:** 1 School of Pharmacy, College of Pharmacy, Kaohsiung Medical University, Kaohsiung 807, Taiwan; shioulingli1211@gmail.com (S.-L.L.); cindy828204@gmail.com (S.-S.Y.); 2 Graduate Institute of Natural Products, College of Pharmacy, Kaohsiung Medical University, Kaohsiung 807, Taiwan; duncanwu762001@gmail.com; 3 Graduate Institute of Natural Products, College of Medicine, Chang Gung University, Taoyuan 333, Taiwan; htl@mail.cgu.edu.tw; 4 Research Center for Industry of Human Ecology, Research Center for Chinese Herbal Medicine, and Graduate Institute of Health Industry Technology, College of Human Ecology, Chang Gung University of Science and Technology, Taoyuan 333, Taiwan; 5 Department of Anesthesiology, Chang Gung Memorial Hospital, Taoyuan 333, Taiwan; 6 Botanical Drug Technology Division, Biomedical Technology and Device Research Laboratories, Industrial Technology Research Institute, Hsinchu 300, Taiwan; chuhung.lin@gmail.com; 7 Drug Development and Value Creation Research Center, Kaohsiung Medical University, Kaohsiung 807, Taiwan; 8 Department of Medical Research, Kaohsiung Medical University Hospital, Kaohsiung 807, Taiwan

**Keywords:** *Machilus japonica* var. *kusanoi*, lauraceae, butanolide, lignan, anti-inflammatory activity

## Abstract

In a series of anti-inflammatory screenings of lauraceous plants, the methanolic extract of the leaves of *Machilus japonica* var. *kusanoi* (Hayata) J.C. Liao showed potent inhibition on both superoxide anion generation and elastase release in human neutrophils. Bioassay-guided fractionation of the leaves of *M. japonica* var. *kusanoi* led to the isolation of twenty compounds, including six new butanolides, machinolides A–F (**1**–**6**), and fourteen known compounds (**7**–**20**). Their structures were characterized by 1D and 2D NMR, UV, IR, CD, and MS data. The absolute configuration of the new compounds were unambiguously confirmed by single-crystal X-ray diffraction analyses (**1**, **2**, and **3**) and Mosher’s method (**4**, **5**, and **6**). In addition, lignans, (+)-eudesmin (**11**), (+)-methylpiperitol (**12**), (+)-pinoresinol (**13**), and (+)-galbelgin (**16**) exhibited inhibitory effects on *N*-formyl-methionyl-leucyl-phenylalanine/cytochalasin B (fMLP/CB)-induced superoxide anion generation in human neutrophils with IC_50_ values of 8.71 ± 0.74 μM, 2.23 ± 0.92 μM, 6.81 ± 1.07 μM, and 7.15 ± 2.26 μM, respectively. The results revealed the anti-inflammatory potentials of Formosan *Machilus japonica* var. *kusanoi.*

## 1. Introduction

Neutrophils play an important role in the human body against infections [1]. In response to immune stimulation, activated neutrophils generate a series of cytotoxic substances, such as the superoxide anion (O_2_^•−^), a precursor of other ROS, granule proteases, and bioactive lipids. The superoxide anion is known to cause damage to cells and tissues, stimulate macrophages, and trigger a cascade of inflammatory pathways [2]. Neutrophil elastase is one of the serine proteases stored in large amounts in neutrophil granules and is involved in the nonoxidative pathway of the intracellular and extracellular immune response [3]. Neutrophil elastase is stimulated by neutrophils and causes the destruction of tissue in chronic inflammatory disease [2]. Besides, the persistent overexpression of neutrophils is involved in various conditions, such as rheumatoid arthritis, asthma, psoriasis, and ischemic heart disease.

Lauraceous plants are a dominant family in South and East Asia, consisting of aromatic trees and shrubs. They stand out, resulting in its economic benefits and diverse bioactivities. A previous investigation showed that some lauraceous plants exhibit bioactivities, such as cytotoxicity, anti-tuberculosis, anti-inflammatory, and antiplatelet activities [4]. Recently, we completed the anti-inflammatory screening of 174 methanolic extracts from 60 Taiwanese lauraceous plants. Among the screening results, the methanolic extract of the leaves of *Machilus japonica* var. *kusanoi* showed potent anti-inflammatory activity on both superoxide anion generation and elastase release in human neutrophils.

The *Machilus* genus comprises about 100 species with accepted names, mainly distributed in East Asia [5]. Previous studies of *Machilus* species identified various classes of chemical constituents, such as lignans, flavonoids, and terpenoids [4]. *M. japonica* var. *kusanoi* is a large evergreen tree endemic to Taiwan and is distributed in broad-leaved forests from lowlands up to 1400 m throughout the island [5]. Few investigations of *M. japonica* var. *kusanoi* have been published before. Only ten compounds were isolated from this plant [6,7,8], and only antimicrobial along with anti-α-glucosidase activity of this plant have been found previously [8,9]. Based on anti-inflammatory screening results and the rare investigation of the leaves from *M. japonica* var. *kusanoi*, the aims of this study are the isolation of components from the leaves of *M. japonica* var. *kusanoi* and the evaluation of their anti-inflammatory effects.

## 2. Results

After anti-inflammatory assay-guided fractionation of the leaves of *M. japonica* var. *kusanoi*, we successfully isolated six new butanolides (**1**−**6**) (Figure 1) and 14 known compounds (**7**–**20**) (Supplementary Materials, Figure S1). The phytochemical spectra of compounds **1** to **6** are available in the Supplementary Materials, Figures S2–S57. In particular, Mosher’s method and X-ray crystallographic analysis were applied to determine the absolute configuration of the new compounds. Moreover, anti-inflammatory effects of isolates on neutrophil pro-inflammatory responses were evaluated by the suppression of *N*-formyl-methionyl-leucyl-phenylalanine/cytochalasin B (fMLP/CB)-induced superoxide anion (O_2_^•−^) generation and elastase release. The structure identification of the new compounds and anti-inflammatory activity results are illustrated below.

**Figure 2:**
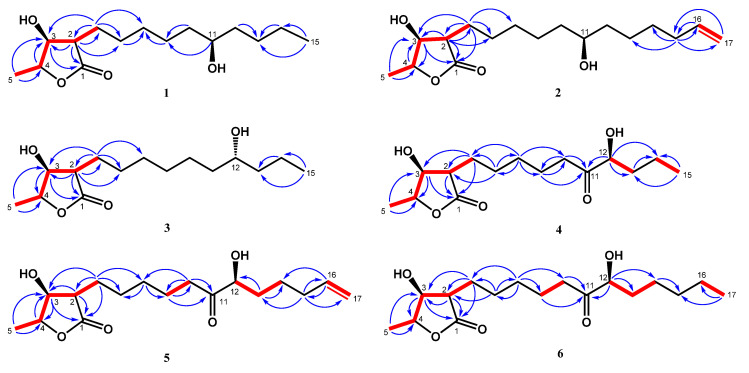
Key ^1^H-^1^H COSY (**━**) and HMBC (H→C) correlations of machinolides A–F (**1**–**6**).

**Figure 3:**
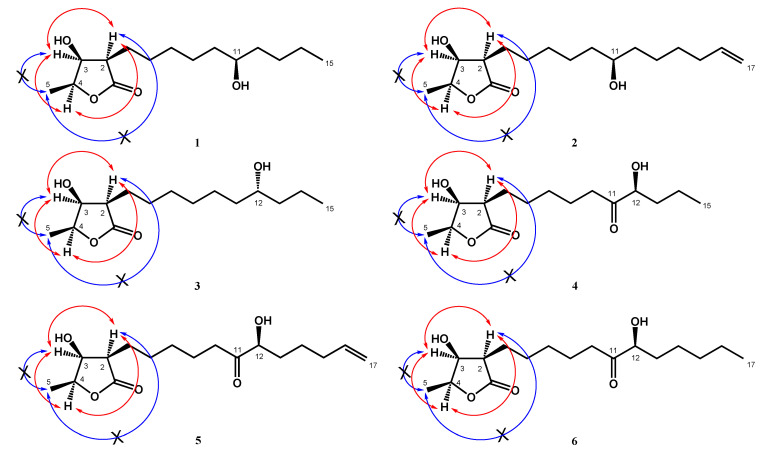
NOESY (H↔H) correlations of machinolides A–F (**1**–**6**).

**Figure 4:**
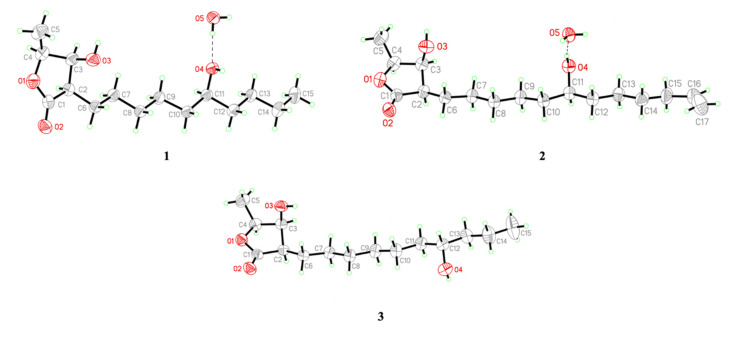
Perspective drawing of X-ray structures of machinolides A–C (**1**–**3**).

Compound **1** was obtained as colorless needles. Its molecular formula was determined as C_15_H_28_O_4_ from high-resolution electrospray ionization mass spectroscopy (HRESIMS) data (*m*/*z* 295.18923 [M + Na]^+^ (calcd. for 295.18853)), implying two degrees of unsaturation. The infrared spectroscopy (IR) spectrum showed typical absorptions of C=O (1736 cm^−1^) for *γ*-lactone and hydroxy groups (3433 cm^−1^). The ^1^H-NMR spectrum of **1** displayed signals of three oxymethines at *δ*_H_ 3.59 (1H, m, H-11), 4.45 (1H, qd, *J* = 6.6, 3.2 Hz, H-4), and 4.31 (1H, dd, *J* = 4.8, 3.2 Hz, H-3), two methyl groups including one doublet methyl group at *δ*_H_ 1.43 (3H, d, *J* = 6.6 Hz, H-5) and one triplet methyl group at *δ*_H_ 0.91 (3H, t, *J* = 7.0 Hz, H-15), and alkyl side chains at *δ*_H_ 1.66 (1H, m, H-6b), *δ*_H_ 1.82 (1H, m, H-6a), and *δ*_H_ 1.26~1.47 (14H, m, H-7~H-10, H-12~H-14) (Table 1). The *γ*-lactone was confirmed by IR spectrum, the ^1^H-^1^H correlation spectroscopy (COSY) correlations between H-2/H-3/H-4/H-5 and the heteronuclear multiple bond correlation (HMBC) between H-2/C-1 (*δ* 177.5), C-3 (*δ* 71.2), H-3/C-1, and H-4/C-3 (Figure 2). The doublet methyl group (C-5) was connected to C-4, based on the COSY correlations between H-5/H-4, and HMBC correlations between H-5/C-3, C-4 (*δ* 78.8) (Figure 2). The HMBC showed correlations H-6/C-2 (*δ* 47.6), C-3, C-7 (*δ* 27.5), and C-8 (*δ* 29.4), which supported that the alkyl chain was located at C-2 (Figure 2). The key correlations in the nuclear Overhauser enhancement spectroscopy (NOESY) spectrum (H-2 showed correlation with H-3, H-4, and no correlation with H-5; H-3 showed correlation with H-4 and no correlation with H-5) confirmed that H-2, H-3, and H-4 were in the same phase (Figure 3). However, a remaining hydroxy group (*δ*_C_ 71.9) was located at a position of the alkyl chain which cannot be determined by NMR spectrum. Finally, the location of the remaining hydroxy group and the absolute configuration of **1** was further confirmed by single-crystal X-ray diffraction (Figure 4). The results proved that the stereochemistry of **1** should be shown as 2*R*,3*S*,4*S*,11*R*-form in the Oak Ridge thermal ellipsoid plot program (ORTEP) diagram. Thus, compound **1** was elucidated and named machinolide A.

Compound **2** was isolated as colorless needles. Its molecular formula was established as C_17_H_30_O_4_ by HREIMS data (*m*/*z* 321.20370 [M + Na]^+^ (calcd. for 321.20363)). The ^1^H-NMR spectrum of **2** was similar to that of **1**, except for the presence of a terminal double bond at *δ*_H_ 5.81 (1H, ddt, *J* = 17.1, 10.2, 6.6 Hz, H-16), 4.94 (1H, ddt, *J* = 10.2, 3.3, 1.5 Hz, H-17b), and 5.00 (1H, ddt, *J* = 17.1, 3.3, 1.5 Hz, H-17a) in **2** (Table 1). Comparison of ^13^C-NMR spectrum of **2** and **1** also supported the presence of a terminal double bond [*δ*_C_ 138.9 (C-16), 114.4 (C-17)] in **2**. The HMBC correlations between H-15/C-16, C-17 and H-16, H-17/C-15 (*δ* 33.7) were further confirmed that the terminal double bond was located at C-16 and C-17 (Figure 2). The NOESY correlations of **2** were similar to those of **1**, indicating that H-2, H-3 and H-4 were in the same phase in **2** (Figure 3). The absolute configuration of **2** was confirmed by single-crystal X-ray diffraction and assigned as 2*R*,3*S*,4*S*,11*S*-form (Figure 4). According to the above data, the structure of **2** was determined and named machinolide B.

Compound **3** was yielded as colorless needles and assigned the molecular formula C_15_H_28_O_4_ through analysis of its HRESIMS data (*m*/*z* 273.20656 [M + H]^+^ (calcd. for 273.20658). All the spectra of **3** were similar to those of **1**. However, electron ionization mass spectra (EIMS) showed the different fragments between **3** (*m*/*z* 215 (56), 186 (37)) and **1** (*m*/*z* 229 (39), 200 (24)), which suggests that the position of the hydroxy group in the alkyl chain was different. The hydroxy group of **3** was located at C-12 and the absolute configuration of **3** was assigned as 2*R*,3*S*,4*S*,12*R*-form, which were both determined by single-crystal X-ray diffraction (Figure 4). Therefore, compound **3** was named machinolide C, and its structure was further confirmed by COSY and HMBC experiments (Figure 2).

**Figure 5:**
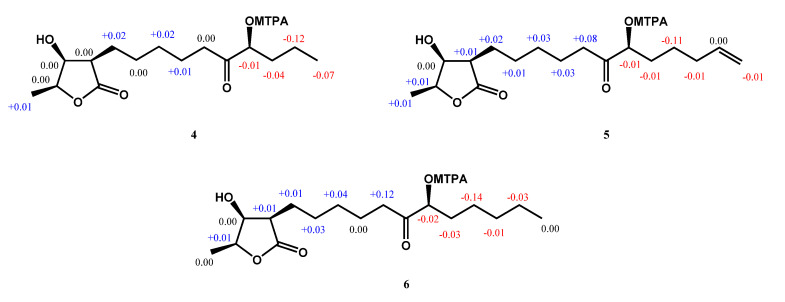
Results with the modified Mosher’s method (Δ*δ*_S–R_) of machinolides D–F (**4**–**6**).

Compound **4** was obtained as a colorless oil. The ESIMS analysis of **4** showed the [M+H]^+^ ion at *m*/*z* 287, in agreement with the molecular formula of C_15_H_26_O_5_, as confirmed by HRESIMS. Compound **4** had similar IR and ^1^H-NMR spectra to those of **3**, except for the presence of a ketone group at C-11 (*δ* 212.6) in the ^13^C-NMR spectrum (Table 2). The HMBC correlation between H-9, H-10, H-12/C-11, and H-12/C-11, C-13, C-14 supported the position of the ketone group and hydroxy group at C-11 and C-12, respectively (Figure 2). The planar structure of **4** was decided. The CD spectrum of **4** showed a negative cotton effect at 219.5 nm, which was similar to malleastrumolide A [10]. Thus, the absolute configuration of C-2 was determined as *R*-form. The NOESY correlations between H-2/H-3, H-2/H-4, and H-3/H-4 confirmed that H-2, H-3, and H-4 were in the same phase (Figure 3). Hence, the absolute configuration of **4** was determined as 2*R*,3*S*,4*S*-form. Based on the ^13^C-NMR-based empirical rules, the chemical shifts of C-3 and C-4 in **4** were similar to those of 2*R*,3*S*,4*S*-form compounds in the literature [11]. According to these two pieces of evidence, the absolute configuration of C-2, C-3, and C-4 in **4** was established to be 2*R*,3*S*,4*S*-form. The absolute configuration of C-12 was determined by Mosher’s method [12]. Based on the Δ*δ* values of the (*S*)-MTPA and (*R*)-MTPA esters in chloroform-*d*_1_, the absolute configuration of C-12 was established as *S*-form (Figure 5). Accordingly, the absolute configuration of **4** was defined as 2*R*,3*S*,4*S*,12*S*. The structure of **4** was confirmed and named machinolide D.

Compound **5** was isolated as a colorless oil. The ESIMS (*m*/*z* 313 [M + H]^+^) and HRESIMS (*m*/*z* 335.18295 [M + Na]^+^ (calcd. for 335.18290)) data were used to establish the molecular formula of compound **5** as C_17_H_28_O_5_. The ^1^H-NMR spectrum of **5** was similar to that of **4**, except for the presence of a terminal double bond at *δ*_H_ 5.78 (1H, ddt, *J* = 17.2, 10.4, 6.8 Hz, H-16), 4.98 (1H, m, H-17b), and 5.03 (1H, m, H-17a) in **4** (Table 2). The HMBC correlation between H-15/C-16, C-17, H-16/C-15, and the COSY correlation between H-16/H-17 supports the presence of a terminal double bond (Figure 2). The CD spectrum (a negative cotton effect at 217.5 nm) and NOESY correlation (Figure 3) of **5** were also similar to **4**. Moreover, in accordance with the ^13^C-NMR-based empirical rules [11], the chemical shifts of C-3 and C-4 in **5** were similar to those of 2*R*,3*S*,4*S*-form compounds in the previous data [11], showing that the absolute configuration of **6** was 2*R*,3*S*,4*S*-form. The absolute configuration of C-12 in **5** was established as *S*-form by Mosher’s method (Figure 5). On the basis of the above results, the structure and absolute configuration of **5** were determined and named machinolide E.

Compound **6** was purified as a colorless oil. Its molecular formula of C_17_H_30_O_5_, two protons more than **5**, was determined by EIMS (*m*/*z* 315 [M + H]^+^) and HRESIMS *m*/*z* 337.19849 [M + Na]^+^ (calcd. for 337.19855). The difference between **6** and **5** is that the terminal double bond in **5** is replaced by the ethyl group in **6**. The HMBC correlations between H-17/C-15, C-16, and the COSY correlation between H-16/H-17 (Figure 2) also supported the presence of the ethyl group. The absolute configuration of **6** was elucidated as 2*R*,3*S*,4*S*,*12S*-form by the CD spectrum, NOESY correlation, and Mosher’s method. As determined by the above observations, the structure of **6** was elucidated as a new compound and named machinolide F.

By comparison of the experiments and reported spectroscopic data ([α]_D_, UV, IR, NMR, and MS), known compounds were identified as one apocarotenoid: blumenol A (**7**) [13], one benzenoid: amisbenzoic acid (**8**) [14], one chlorophyll: pheophytin a (**9**) [15], one coumarin: isofraxidin (**10**) [16], six lignans: (+)-eudesmin (**11**) [17], (+)-methylpiperitol (**12**) [18], (+)-pinoresinol (**13**) [19], (+)-syringaresinol (**14**) [20], (2*S*,5*S*)-diveratryl-(3*R*,4*S*)-dimethyltetrahydrofuran (**15**) [21], and (+)-galbelgin (**16**) [22], three sesquiterpenoids: β-eudesmol (**17**) [23], caryophyllene oxide (**18**), and clovane-2α,9β-diol (**19**) [24], and one steroid: β-sitosterol (**20**) [25].

In this study, eight isolates present in sufficient amounts (**1**, **2**, **3**, **6**, **11**–**13**, and **16**) were evaluated for an inhibitory effect on fMLP/CB-induced superoxide anion (O_2_^•−^) generation and elastase release (Table 3). (+)-Eudesmin (**11**), (+)-methylpiperitol (**12**), (+)-pinoresinol (**13**), and (+)-galbelgin (**16**) displayed inhibitory activity on superoxide anions in fMLP/CB-stimulated human neutrophils with IC_50_ values of 8.71 ± 0.74 μM, 2.23 ± 0.92 μM, 6.81 ± 1.07 μM, and 7.15 ± 2.26 μM, respectively. LY294002 (Sigma-Aldrich), a potent phosphatidylinositol 3-kinase (PI3K) inhibitor, was used as a positive control to inhibit O_2_^•−^ generation and elastase release, with IC_50_ values of 2.17 ± 0.53, and 6.38 ± 1.72 μM, respectively.

## 3. Discussion

Inflammation is triggered by infection or tissue injury. In our series of anti-inflammatory screenings of lauraceous plants, the leaves of *M. japonica* var. *kusanoi* stand out as a research candidate. Focusing on the anti-inflammatory activity results in this paper, the lignans, (+)-eudesmin (**11**), (+)-methylpiperitol (**12**), (+)-pinoresinol (**13**), and (+)-galbelgin (**16**) exhibited inhibitory activities on superoxide anion generation. (+)-Methylpiperitol (**12**) showed better anti-inflammatory activity than (+)-eudesmin (**11**), suggesting the methylenedioxy group may enhance the anti-inflammatory activity. (+)-Methylpiperitol (**12**) exhibited similar anti-inflammatory activity as (+)-pinoresinol (**13**), indicating the replacement of the methoxy group may not influence anti-inflammatory activity. The results suggested that the furofuran-type lignan containing a methylenedioxy group showed the best anti-inflammatory activity in this study. More importantly, this is the first report on the anti-inflammatory activity of *M. japonica* var. *kusanoi*.

Butanolides (γ-butyrolactones) are four-carbon heterocyclic lactone ring structures reported from some specific families (Myristicaceae [26], Meliaceae [10], Actinomycetes [27,28,29,30,31]), especially in Lauraceae plants (*Machilus* sp. [32,33,34], *Lindera* sp. [35,36], *Litsea* sp. [37], *Cinnamomum* sp. [38,39,40], *Persea* sp. [41]). The characteristic butanolides in Lauraceae plants contain an alkyl side chain group at C-2, a hydroxy group at C-3, and one methyl group at C-4, with or without a double bond between C-2/C-3 and C-2/C-6. In this report, six new compounds, machinolides A–F (**1**–**6**), were butanolide compounds without a double bond between C-2/C-3 or C-2/C-6. This type of butanolide has not been isolated from *Machilus* before, which might improve our understanding of secondary metabolites from *Machilus* species. The chemical results can contribute to the chemotaxonomy of *Machilus* species.

Although the potency of the lignans exhibiting anti-inflammatory activity in this study was similar to bioactive lignans described in the literature [42], it is worth noting that most of the lignans with anti-inflammatory activity in this study have not been reported previously. Besides, there are no anti-inflammatory medicines act via inhibiting superoxide anion and neutrophil elastase. The research shows some lead compounds and will help develop novel anti-inflammatory drugs.

## 4. Materials and Methods

### 4.1. General Experiment Procedures

Optical rotations were measured on a Jasco P-2000 polarimeter (Jasco, Kyoto, Japan), and IR spectra (ATR) were acquired with a Jasco FT/IR-4600 spectrometer. We recorded 1D (^1^H, ^13^C, DEPT) and 2D (COSY, NOESY, HSQC, HMBC) NMR spectra on a Varian Germini-2000 spectrometer (Varian, Inc. Vacuum Technologies, Lexington, MA, USA) operated at 200 (1H) and 50 MHz (^13^C), a Varian Unityplus-400 spectrometer (Varian, Inc. Vacuum Technologies, Lexington, MA, USA) operated at 400 (^1^H) and 100 MHz (^13^C), a Varian Mercuryplus-400 spectrometer (Varian, Inc. Vacuum Technologies, Lexington, MA, USA) operated at 400 (^1^H) and 100 MHz (^13^C), and a Varian VNMRS-600 spectrometer (Varian, Inc. Vacuum Technologies, Lexington, MA, USA) operated at 600 (^1^H) and 150 MHz (^13^C). Low-resolution mass spectra were obtained with POLARIS Q Thermo Finnigan (Thermo Fisher Scientific, Chicago, IL, USA), Waters ZQ 4000 (Waters, Milford, MA, USA), and VG Quattro GC/MS/MS/DS (Waters, Milford, MA, USA) mass spectrometers. EIMS were taken on a JEOL JMS-700 mass spectrometer (JEOL, Tokyo, Japan). HRESIMS were recorded on a Bruker APEX II mass spectrometer (Bruker, Karlsruhe, Germany) and VARIAN 901-MS (Varian, CA, USA). Silica gel (70–230 and 230–400 mesh; Silicycle, QC, Canada) was used for column chromatography (CC), and silica gel 60 F254 (Merck, Darmstadt, Germany) and RP-18 F254S (Merck, Darmstadt, Germany) were used for thin layer chromatography (TLC) and preparative TLC, respectively, visualized with a Ce_2_(SO_4_)_3_ aqueous solution. Further purification was performed by medium-performance liquid chromatography (MPLC; ceramic pump: VSP-3050; EYELA, Kyoto, Japan).

### 4.2. Plant Material

The leaves of *Machilus japonica* var. *kusanoi* (Hayata) J.C. Liao were collected in March 2018 in Mudan Township, Pingtung County, Taiwan, and identified by I.-S.C. A voucher specimen (Chen 5480) was deposited with the herbarium of the College of Pharmacy, Kaohsiung Medical University, Kaohsiung, Taiwan.

### 4.3. Extraction and Isolation

Dried leaves (5.8 kg) of *M. japonica* var. *kusanoi* were extracted at room temperature with methanol (MeOH) (30 L) three times to yield a MeOH extract (730 g). The MeOH extract was suspended in water and partitioned with ethyl acetate (EtOAc) to give a water layer (265.4 g), EtOAc layer (390 g), and precipitate (72 g). The EtOAc layer (390 g) was taken and 100 g were subjected to column chromatography (silica gel; *n*-hexane/EtOAc 100/0 to 0/100 EtOAc, then washed with 100% acetone and 100% methanol) to yield six fractions (Fr. 1–6). Fr. 3 (15.1 g) was subjected to open column (silica gel; *n*-hexane/acetone 6/1 to 2/1, column size: 3 × 70 cm) to yield 13 fractions (Fr.3-1–3-13). Fr. 3-9 was subjected to MPLC (RP-18; water/methanol 1:1; column size: 1.5 × 30 cm) to give seven fractions (Fr. 3-9-1–3-9-7). Fr. 3-9-1 was subjected to MPLC (silica gel; *n*-hexane/CH_2_Cl_2_/EtOAc 2/2/1 to 1/1/1; column size: 1 × 30 cm) to afford 14 fractions (Fr. 3-9-1-1–3-9-1-14) and compound **7** (30.5 mg). Fr. 3-9-1-11 was subjected to MPLC (silica gel; *n*-hexane/CH_2_Cl_2_/methanol 15/20/1; column size: 1 × 30 cm) to produce compound **6** (14.0 mg). Fr. 3-9-2 was subjected to MPLC (silica gel; *n*-hexane/CH_2_Cl_2_/EtOAc 2/2/1; column size: 1 × 30 cm) to obtain 10 fractions (Fr. 3-9-2-1–3-9-2-10). Fr. 3-9-2-9 was subjected to MPLC (silica gel; *n*-hexane/CH_2_Cl_2_/EtOAc 2/1/1; column size: 1 × 30 cm) to afford compound **11** (10.7 mg). Fr. 3-9-3 was subjected to MPLC (silica gel; *n*-hexane/acetone 2/1; column size: 1 × 30 cm) to furnish compound **19** (1.0 mg). Fr. 3-10 was separated with Sephadex LH-20 (column size: 3 × 70 cm) and eluted with methanol to provide seven fractions (3-10-1–3-10-7). Fr. 3-10-2 was subjected to MPLC (silica gel; H_2_O/methanol 1/1 to 2/3; column size: 1.5 × 30 cm) to gain 14 fractions (Fr. 3-10-2-1–3-10-2-14). Fr. 3-10-2-4 was subjected to MPLC (silica gel; CH_2_Cl_2_/EtOAc 3/1; column size: 1 × 30 cm) to obtain compound **4** (5.3 mg). Fr. 3-10-2-7 was subjected to MPLC (silica gel; CH_2_Cl_2_/EtOAc 3/1; column size: 1 × 30 cm) to produce compound **5** (0.9 mg). Fr. 3-10-2-13 was subjected to MPLC (silica gel; CH_2_Cl_2_/EtOAc 4/1; column size: 1 × 30 cm) to yield compound **2** (2.3 mg). Fr. 3-10-2-15 was subjected to MPLC (silica gel; CH_2_Cl_2_/acetone 15/1; column size: 1 × 30 cm) to afford five fractions (Fr. 3-10-2-15-1–3-10-2-15-5). Fr. 3-10-2-15-3 was subjected to MPLC (silica gel; CH_2_Cl_2_/EtOAc 3/1; column size: 1 × 30 cm) to give compounds **3** (2.8 mg) and **1** (5.9 mg). Fr. 3-10-4 was subjected to MPLC (silica gel; H_2_O/methanol 2/3; column size: 1 × 30 cm) to produce 11 fractions (Fr. 3-10-4-1–3-10-4-11). Fr. 3-10-4-2 was subjected to MPLC (silica gel; *n*-hexane/CH_2_Cl_2_/EtOAc 2/2/1; column size: 1 × 30 cm) to furnish compound **8** (0.5 mg). Fr. 3-10-5 was subjected to MPLC (RP-18; water/methanol 2/1 to 1/1; column size: 1 × 30 cm) to give compound **10** (0.3 mg). Fr. 3-7 was subjected to MPLC (silica gel; *n*-hexane/EtOAc 3/1 to 3/2; column size: 1.5 × 30 cm) to give five fractions (Fr. 3-7-1–3-7-5). Fr. 3-7-3 was subjected to MPLC (RP-18; water/methanol 1/1 to 1/3; column size: 1.5 × 30 cm) to provide nine fractions (Fr. 3-7-3-1–3-7-3-9). Fr. 3-7-3-5 was subjected to MPLC (silica gel; *n*-hexane/CH_2_Cl_2_/EtOAc 4/2/1; column size: 1 × 30 cm) to afford 10 fractions (Fr. 3-7-3-5-1–3-7-3-5-10). Fr. 3-7-3-5-4 was subjected to MPLC (silica gel; *n*-hexane/CH_2_Cl_2_/EtOAc 1/3/0.3; column size: 1 × 30 cm) to give compound **12** (4.6 mg). Fr. 3-7-3-7 was subjected to MPLC (silica gel; *n*-hexane/CH_2_Cl_2_/EtOAc 4/2/1; column size: 1 × 30 cm) to produce nine fractions (Fr. 3-7-3-7-1–3-7-3-7-9). Fr. 3-7-3-7-2 was subjected to MPLC (silica gel; *n*-hexane/CH_2_Cl_2_/EtOAc 6/2/1; column size: 1 × 30 cm) to obtain compounds **15** (0.2 mg) and **16** (2.5 mg). Fr. 3-7-3-7-6 was subjected to MPLC (RP-18; water/methanol 1/3; column size: 1 × 30 cm) to give compound **17** (2.9 mg). Fr. 3-11 was separated with Sephadex LH-20 (column size: 3 × 70 cm) and eluted with methanol to provide 11 fractions (3-11-1–3-11-11). Fr. 3-11-7 was subjected to MPLC (RP-18; water/acetone 3/2; column size: 1 × 30 cm) to give compound **13** (0.5 mg). Fr. 2 was subjected to column chromatography (silica gel; *n*-hexane/CH_2_Cl_2_/acetone 17/1/1 to 10/1/1) to yield ten fractions (Fr. 2-1–2-10). Fr. 2-4 was subjected to MPLC (silica gel; *n*-hexane/acetone 40/1 to 20/1; column size: 2 × 30 cm) to yield nine fractions (Fr. 2-4-1–2-4-9). Fr. 2-4-3 was subjected to MPLC (RP-18; water/acetone 1/5; column size: 1.5 × 30 cm) to produce nine fractions (Fr. 2-4-3-1–2-4-3-9). Fr. 2-4-3-3 was subjected to MPLC (silica gel; *n*-hexane/acetone 40/0.5; column size: 1 × 30 cm) to afford eight fractions (Fr. 2-4-3-3-1–2-4-3-3-8). Fr. 2-4-3-3-3 was subjected to HPLC to obtain two fractions (Fr. 2-4-3-3-3-1–2-4-3-3-3-2). Fr. 2-4-3-3-3-2 was further separated with prep. RP-18 TLC (water/acetonitrile = 1/10) to give compound **18** (1.9 mg). Fr. 2-7 was subjected to column chromatography (silica gel; *n*-hexane/CH_2_Cl_2_/acetone 20/4/1 to 12/4/1) to produce eight fractions (Fr. 2-7-1–2-7-8). Fr. 2-7-2 was subjected to MPLC (silica gel; *n*-hexane/CH_2_Cl_2_/acetone 12/4/1; column size: 2 × 30 cm) to give compound **20** (1.6 g). Fr. 2-8 was subjected to column chromatography (silica gel; *n*-hexane/CH_2_Cl_2_/acetone 16/16/1 to 8/16/1) to afford 14 fractions (Fr. 2-8-1–2-8-14). Fr. 2-8-7 was subjected to MPLC (silica gel; *n*-hexane/acetone 6/1; column size: 1.5 × 30 cm) to produce seven fractions (Fr. 2-8-7-1–2-8-7-7). Fr. 2-8-7-5 was subjected to MPLC (silica gel; *n*-hexane/CH_2_Cl_2_/acetone 16/16/1; column size: 1 × 30 cm) to produce compound **9** (15.2 mg). Fr. 4 was subjected to column chromatography (silica gel; *n*-hexane/acetone 5/1 to 3/1) to yield six fractions (Fr. 4-1–4-6). Fr. 4-5 was separated with Sephadex LH-20 (column size: 3 × 70 cm) and eluted with methanol to provide six fractions (4-5-1–4-5-6). Fr. 4-5-4 was subjected to MPLC (RP-18; water/methanol 1/1; column size: 1.5 × 30 cm) to produce six fractions (Fr. 4-5-4-1–4-5-4-6). Fr. 4-5-4-1 was subjected to MPLC (RP-18; water/methanol 1/1; column size: 1 × 30 cm) to afford four fractions (Fr. 4-5-4-1-1–4-5-4-1-4). Fr. 4-5-4-1-1 was subjected to MPLC (silica gel; *n*-hexane/CH_2_Cl_2_/acetone 2/2/1; column size: 1 × 30 cm) to give compound **14** (1.6 mg). (Supplementary Materials, Figure S58)

#### 4.3.1. Machinolide A (**1**)

Colorless needles; ${\left[\alpha \right]}_{D}^{25}$ −29.6 (*c* 0.30, MeOH); IR *ν*_max_ (ATR): 3433 (OH), 1736 (γ-lactone) cm^−1^; ^1^H-NMR and ^13^C-NMR (Table 1); ESIMS *m*/*z* 273 [M + H]^+^; EIMS *m*/*z* (rel. int.): 254 ([M − H_2_O]^+^, 3), 215 (56), 186 (37), 129 (99), 57 (100); HRESIMS *m*/*z* 295.18923 [M + Na]^+^ (calcd. for C_15_H_28_NaO_4_, 295.18853).

#### 4.3.2. Machinolide B (**2**)

Colorless needles; ${\left[\alpha \right]}_{D}^{25}$ −42.1 (*c* 0.092, MeOH); IR *ν*_max_ (ATR): 3436 (OH), 1737 (γ-lactone) cm^−1^; ^1^H-NMR and ^13^C-NMR (Table 1); ESIMS *m*/*z* 299 [M + H]^+^; HRESIMS *m*/*z* 321.20370 [M + Na]^+^ (calcd. for C_17_H_30_NaO_4_, 321.20363).

#### 4.3.3. Machinolide C (**3**)

Colorless needles; ${\left[\alpha \right]}_{D}^{25}$ −42.0 (*c* 0.14, MeOH); IR *ν*_max_ (ATR): 3321 (OH), 1743 (γ-lactone) nm; ^1^H-NMR and ^13^C-NMR (Table 2); ESIMS *m*/*z* 273 [M + H]^+^; EIMS *m*/*z* (rel. int.): 254 ([M − H_2_O]^+^, 3), 229 (39), 200 (24), 129 (80), 57 (100); HRESIMS *m*/*z* 273.20656 [M + H]^+^ (calcd. for C_15_H_29_O_4_, 273.20658).

#### 4.3.4. Machinolide D (**4**)

Colorless oil; ${\left[\alpha \right]}_{D}^{27}$ −42.5 (*c* 0.25, MeOH); IR *ν*_max_ (ATR): 3437 (OH), 1752 (γ-lactone), 1708 (C=O) cm^−1^; CD *λ*_ext_ (MeOH) (Δ*ε*): 280 (+15.43), 219.5 (−54.44) nm; ^1^H-NMR and ^13^C-NMR (Table 3); ESIMS *m*/*z* 287 [M + H]^+^; HRESIMS *m*/*z* 309.16726 [M + Na]^+^ (calcd. for C_15_H_26_NaO_5_, 309.16725).

#### 4.3.5. Machinolide E (**5**)

Colorless oil; ${\left[\alpha \right]}_{D}^{26}$ −35.4 (*c* 0.145, MeOH); IR *ν*_max_ (ATR): 3445 (OH), 1748 (γ-lactone), 1713 (C=O) cm^−1^; CD *λ*_ext_ (MeOH) (Δ*ε*): 280.5 (+17.23), 217.5 (−52.40) nm; ^1^H-NMR and ^13^C-NMR (Table 3); ESIMS *m*/*z* 313 [M + H]^+^; HRESIMS *m*/*z* 335.18295 [M + Na]^+^ (calcd. for C_17_H_28_NaO_5_, 335.18290).

#### 4.3.6. Machinolide F (**6**)

Colorless oil; ${\left[\alpha \right]}_{D}^{20}$ −39.4 (*c* 0.85, MeOH); IR *ν*_max_ (ATR): 3440 (OH), 1751 (γ-lactone), 1705 (C=O) cm^−1^; CD *λ*_ext_ (MeOH) (Δ*ε*): 280.5 (+17.34), 217.5 (−52.74) nm; ^1^H-NMR and ^13^C-NMR (Table 3); ESIMS *m*/*z* 315 [M + H]^+^; HRESIMS *m*/*z* 337.19849 [M + Na]^+^ (calcd. for C_17_H_30_NaO_5_, 337.19855).

### 4.4. X-Ray Crystallographic Data for Machinolide A (***1***), Machinolide B (***2***), and Machinolide C (***3***)

The absolute configurations of **1**, **2,** and **3** were determined from data collected on a Bruker D8 VENTURE single-crystal XRD equipped with Oxford Cryostream 800^+^. Crystallographic data for **1**: C_15_H_30_O_5_, *M* = 290.39, size 0.220 × 0.097 × 0.057 mm^3^, orthorhombic, space group *P*2_1_2_1_2_1_, *a* = 4.72807(10) Å, *b* = 12.9141(3) Å, *c* = 27.8178(6) Å, α = β = γ = 90°, *V* = 1698.53(6) Å^3^, *T* = 200(2) K, *Z* = 4, *d*_calcd_ = 1.136 Mg/m^3^, *λ*(Cu Kα) = 1.54178 Å, *F*(000) = 640, reflections collected/independent reflections 9080/3467 [R(int) = 0.0307], final *R* indices *R*_1_ = 0.0331, w*R*_2_ = 0.0928, GOF on F^2^ = 1.046, absolute structure parameter = −0.02(7).

Crystallographic data for **2**: C_17_H_32_O_5_, *M* = 316.42, size 0.397 × 0.052 × 0.036 mm^3^, orthorhombic, space group *P*2_1_2_1_2_1_, *a* = 4.76120(10) Å, *b* = 12.8614(4) Å, *c* = 30.6325(9) Å, α = β = γ = 90°, *V* = 1875.80(9) Å^3^, *Z* = 4, *d*_calcd_ = 1.120 Mg/m^3^, *λ*(Cu Kα) = 1.54178 Å, *F*(000) = 696, reflections collected/independent reflections 10397/3812 [R(int) = 0.0366], final *R* indices *R*_1_ = 0.0568, *wR*_2_ = 0.1537, GOF on F^2^ = 1.036, absolute structure parameter = −0.01 (14).

Crystallographic data for **3**: C_15_H_28_O_4_, *M* = 272.37, size 0.392 × 0.089 × 0.014 mm^3^, orthorhombic, space group *P*2_1_2_1_2_1_, *a* = 4.75710(10) Å, *b* = 9.7931(2) Å, *c* = 35.3857(8) Å, α = β = γ = 90°, *V* = 1648.50(6) Å^3^, *T* = 200(2) K, *Z* = 4, *d*_calcd_ = 1.097 Mg/m^3^, *λ*(Cu Kα) = 1.54178 Å, *F*(000) = 600, reflections collected/independent reflections 15562/3377 [R(int) = 0.0525], final *R* indices *R*_1_ = 0.0431, *wR*_2_ = 0.1152, GOF on F^2^ = 1.037, absolute structure parameter = 0.02(10).

### 4.5. Preparation of (S)-MTPA and (R)-MTPA Esters of ***4a**, **4b**, **5a**, **5b**, **6a***, and ***6b*** from ***4**, **5***, and ***6***

Compound **4** (1.0 mg, 3.5 µmol) and pyridine-*d*_5_ (10.9 µL, 135.4 µmol) was transferred to a vial. The contents of the vial were dissolved in chloroform-*d*_1_ (1090 µL, [4] = 3.5 mM). *R*-(−)-MPTA-Cl (10.9 µL, 58.3 µmol) was added to the vial, the vial was capped and the contents were stirred at room temperature (2–4 h). The (*S*)-MTPA ester (**4a**) was purified by prep. TLC plate (*n*-hexane/EtOAc = 1/1), and its ^1^H-NMR spectra were obtained. The (*R*)-MTPA ester (**4b**) was prepared with (*S*)-MTPA chloride in the same manner. The same method was used to prepare the (*S*)- and (*R*)-MTPA esters of **5a**, **5b**, **6a**, and **6b** (Supplementary Materials, Figures S59–S64).

### 4.6. Superoxide Anion and Elastase Release Assays

The ability of the test compounds to modulate superoxide anion generation and elastase release by neutrophils was evaluated according to the studies published by co-author Professor Tsong-Long Hwang [2,43]. The superoxide generation assay was based on the reduction of ferricytochrome *c* by superoxide dismutase (SOD). Elastase substrate (methoxysuccinyl-Ala-Ala-Pro-Val-p-nitroanilide, 100 μM; Merck) was used to detect elastase release. Elastase level was detected at OD405 nm using a spectrophotometer. PI3K inhibitor LY29002 served as a positive control for the neutrophil assays. All assays were repeated at least three times. Results are presented as mean ± standard error of the mean (SEM). The Student’s *t*-test was used to compare the test compound with a DMSO (0.1%) control. A probability of less than 0.05 was considered significant.

## 5. Conclusions

Six new butanolides, machinolides A–F (**1**–**6**), together with 14 known compounds, were obtained from the leaves of *M. japonica* var. *kusanoi*. The absolute configurations of these new compounds were assigned by their CD spectrum, single-crystal X-ray diffraction analyses, and Mosher’s method. Hence, absolute configurations of all new compounds were determined as 2*R*,3*S*,4*S*-form in a furan ring, and the chiral center in the side chain group was *R*-form in **1** and **3**, and *S*-form in **2**, **4**, **5**, and **6**. Besides, butanolides and lignans were major skeletons in this study. Bioactivity results indicated that lignans could reduce superoxide anion generation in fMLP/CB-stimulated human neutrophils, and the anti-inflammatory activities of those compounds were as potent as compounds in the literature [42]. Furthermore, the structure-activity relationship (SAR) discussion of anti-inflammatory activity compounds indicated that furofuran lignan with methylenedioxy was the most active structure. To our knowledge, this is the first report on anti-inflammatory activity from the leaves of *M. japonica* var. *kusanoi* and the results are helpful to patients with inflammation-related disease.

## Figures and Tables

**Figure 1:**
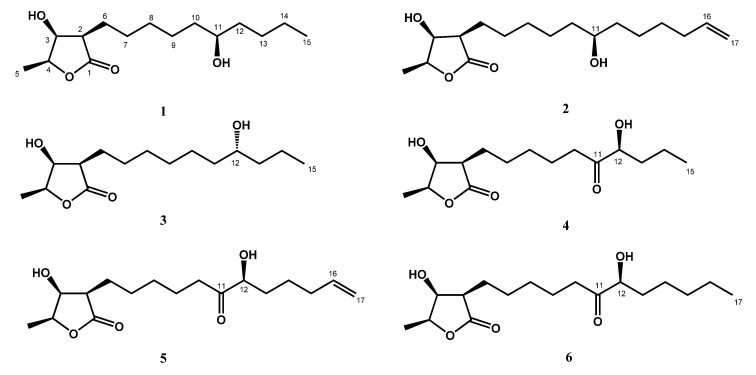
Structures of new compounds **1**–**6**.

**Table 1:** ^1^H and ^13^C-NMR data of machinolides A–C (**1**–**3**).

Position	1 ^a^		2 ^b^		3 ^b^	
	*δ*_H_ (m, *J* in Hz)	*δ*_C_	*δ*_H_ (m, *J* in Hz)	*δ*_C_	*δ*_H_ (m, *J* in Hz)	*δ*_C_
1		177.5		177.4		177.4
2	2.57, dt (9.8, 4.8)	47.6	2.57, dt (10.2, 4.6)	47.6	2.57, dt (8.7, 4.8)	47.6
3	4.31, dd (4.8, 3.2)	71.2	4.31, dd (4.6, 3.2)	71.3	4.31, dd (4.8, 3.2)	71.3
4	4.45, qd, (6.6, 3.2)	78.8	4.45, qd (6.6, 3.2)	78.7	4.45, qd (6.6, 3.2)	78.7
5	1.43, d (6.6)	13.7	1.44, d (6.6)	13.7	1.44, d (6.6)	13.7
6	1.82, m 1.66, m	23.1	1.84, m 1.67, m	23.2	1.84, m 1.65, m	23.3
7	1.26~1.47, m	27.5	1.32~1.51, m	27.6	1.30~1.49, m	27.5
8	1.26~1.47, m	29.4	1.32~1.51, m	29.4	1.30~1.49, m	29.3 ^c^
9	1.26~1.47, m	25.1	1.32~1.51, m	25.1 ^c^	1.30~1.49, m	29.4 ^c^
10	1.26~1.47, m	37.16 ^c^	1.32~1.51, m	37.2 ^d^	1.30~1.49, m	25.5
11	3.59, m	71.9	3.59, m	71.9	1.30~1.49, m	37.4
12	1.26~1.47, m	37.19 ^c^	1.32~1.51, m	37.4 ^d^	3.60, m	71.8
13	1.26~1.47, m	27.8	1.32~1.51, m	25.2 ^c^	1.30~1.49, m	39.7
14	1.26~1.47, m	22.7	1.37, m	28.9	1.30~1.49, m	18.8
15	0.91, t (7.0)	14.0	2.07, m	33.7	0.93, t (7.2)	14.1
16			5.81, ddt (17.1, 10.2, 6.6)	138.9		
17			5.00, ddt (17.1, 3.3, 1.5) 4.94, ddt (10.2, 3.3, 1.5)	114.4		

^a^^1^H (400 MHz, CDCl_3_) and ^13^C-NMR (100 MHz, CDCl_3_). ^b^^1^H (600 MHz, CDCl_3_) and ^13^C-NMR (150 MHz, CDCl_3_). ^c,d^ the data in the same column are interchangeable.

**Table 2:** ^1^H and ^13^C-NMR data of machinolides D–F (**4**–**6**).

Position	4 ^a^		5 ^b^		6 ^b^	
	*δ*_H_ (m, *J* in Hz)	*δ*_C_	*δ*_H_ (m, *J* in Hz)	*δ*_C_	*δ*_H_ (m, *J* in Hz)	*δ*_C_
**1**		177.4		177.3		177.7
**2**	2.55, dt (9.9, 5.0)	47.5	2.56, dt (9.8, 4.5)	47.5	2.54, dt (10.0, 5.0)	47.5
**3**	4.31, dd (5.0, 3.0)	71.3	4.30, br t (4.5)	71.3	4.30, dd (5.0, 3.1)	71.1
**4**	4.45, qd (6.5, 3.0)	78.8	4.54, qd (6.4, 2.9)	78.7	4.44, qd (6.0, 3.1)	79.0
**5**	1.44, d (6.5)	13.7	1.44, d (6.4)	13.7	1.42, d (6.0)	13.7
**6**	1.80, m 1.67, m	23.1	1.84, m 1.67, m	23.1	1.79, m 1.64, m	23.1
**7**	1.35~1.55, m	27.4	1.33~1.58, m	27.4	1.24~1.52, m	27.3
**8**	1.35~1.55, m	29.0	1.33~1.58, m	29.0	1.24~1.52, m	29.0
**9**	1.67, m	23.2	1.67, m	23.2	1.64, m	23.2
**10**	2.40~2.54, m	37.7	2.46, m	37.7	2.46, m	37.7
**11**		212.6		212.4		212.7
**12**	4.17, dd (7.5, 3.9)	76.3	4.17, dd (7.2, 3.6)	76.3	4.15, dd (7.4, 3.8)	76.5
**13**	1.35~1.55, m	35.9	1.84, m 1.33~1.58, m	33.1	1.79, m 1.24~1.52, m	33.7
**14**	1.35~1.55, m	18.2	1.33~1.58, m	24.0	1.24~1.52, m	24.5
**15**	0.95, t (6.9)	13.9	2.09, m	33.3	1.24~1.52, m	31.6
**16**			5.78, ddt (17.2, 10.4, 6.8)	138.1	1.24~1.52, m	22.5
**17**			5.03, m 4.98, m	115.1	0.88, t (6.8)	14.0

^a^^1^H (600 MHz, CDCl_3_) and ^13^C-NMR (150 MHz, CDCl_3_). ^b^^1^H (400 MHz, CDCl_3_) and ^13^C-NMR (100 MHz, CDCl_3_).

**Table 3:** Effect of compounds on superoxide anion generation and elastase release in fMLP/CB-stimulated human neutrophils.

Compound	Superoxide Anion	Elastase Release
	IC_50_ (μM) ^a^	IC_50_ (μM) ^a^
machinolide A (**1**)	>10	>10
machinolide B (**2**)	>10	>10
machinolide C (**3**)	>10	>10
machinolide F (**6**)	>10	>10
(+)-eudesmin (**11**)	8.71 ± 0.74	>10
(+)-methylpiperitol (**12**)	2.23 ± 0.92	>10
(+)-pinoresinol (**13**)	6.81 ± 1.07	>10
(+)-galbelgin (**16**)	7.15 ± 2.26	>10
LY294002 ^b^	2.17 ± 0.53	6.38 ± 1.72

^a^ Concentration necessary for 50% inhibition (IC_50_). ^b^ Positive control.

## Supplementary Materials

Figure S1: Structures of known compounds **7**–**20**, Figures S2–S57: The phytochemical spectra of compounds **1**–**6**. Figure S58: Extraction and isolation of the leaves from *M. japonica* var. *kusanoi*, Figures S59–S64: The phytochemical spectra of compounds **4a**, **4b**, **5a**, **5b**, **6a**, and **6b**. Table S1: Inhibitory effects of crude extracts from the leaves of *M. japonica* var. *kusanoi* on superoxide anion generation and elastase release in fMLP/CB-induced human neutrophils.
