# Supplementary material for: Phytochemical Investigation and Anti-Inflammatory Activity of the Leaves of Machilus japonica var. kusanoi
Source: Molecules. 2020 Sep 10;25(18):4149. doi: 10.3390/molecules25184149 (PMC7570621; doi:10.3390/molecules25184149)
Supplement: Supplementary file 1 [file molecules-25-04149-s001.pdf]

Phytochemical Investigation and Anti-Inflammatory Activity of the Leaves of *Machilus japonica* var. *kusanoi*

Shiou-Ling Li [1,2], Ho-Cheng Wu [2], Tsong-Long Hwang [3,4,5], Chu-Hung Lin [6], Shuen-Shin Yang [1] and Hsun-Shuo Chang [1,2,7,8,*]

[1] School of Pharmacy, College of Pharmacy, Kaohsiung Medical University, Kaohsiung 807, Taiwan; shioulingli1211@gmail.com (S.-L.L.); cindy828204@gmail.com (S.-S.Y.)
[2] Graduate Institute of Natural Products, College of Pharmacy, Kaohsiung Medical University, Kaohsiung 807, Taiwan; duncanwu762001@gmail.com
[3] Graduate Institute of Natural Products, College of Medicine, Chang Gung University, Taoyuan 333, Taiwan; htl@mail.cgu.edu.tw
[4] Research Center for Industry of Human Ecology, Research Center for Chinese Herbal Medicine, and Graduate Institute of Health Industry Technology, College of Human Ecology, Chang Gung University of Science and Technology, Taoyuan 333, Taiwan
[5] Department of Anesthesiology, Chang Gung Memorial Hospital, Taoyuan 333, Taiwan
[6] Botanical Drug Technology Division, Biomedical Technology and Device Research Laboratories, Industrial Technology Research Institute, Hsinchu 300, Taiwan; chuhung.lin@gmail.com
[7] Drug Development and Value Creation Research Center, Kaohsiung Medical University, Kaohsiung 807, Taiwan
[8] Department of Medical Research, Kaohsiung Medical University Hospital, Kaohsiung 807, Taiwan
* Correspondence: hschang@kmu.edu.tw; Tel.: +886-7-312-1101 (ext. 2664)

The List of Supplementary Material

Figure S1. Structures of known compounds 7–20. ········· 5

Figure S2. ^{1}H NMR spectrum of (400 MHz, $CDCl_3$) spectrum of 1 ········· 5

Figure S3. ^{13}C NMR spectrum of (100 MHz, $CDCl_3$) spectrum of 1 ········· 6

Figure S4. DEPT spectrum of 1 ········· 6

Figure S5. COSY spectrum of 1 ········· 7

Figure S6. HSQC spectrum of 1 ········· 7

Figure S7. HMBC spectrum of 1 ········· 8

Figure S8. NOESY spectrum of 1 ········· 8

Figure S9. EIMS spectrum of 1 ········· 9

Figure S10. ESIMS spectrum of 1 ········· 9

Figure S11. HRESIMS spectrum of 1 ········· 10

Figure S12. ^{1}H NMR spectrum of (600 MHz, $CDCl_3$) spectrum of 2 ········· 10

Figure S13. ^{13}C NMR spectrum of (150 MHz, $CDCl_3$) spectrum of 2 ········· 11

Figure S14. DEPT spectrum of 2 ········· 11

Figure S15. COSY spectrum of 2 ········· 12

Figure S16. HSQC spectrum of 2 ········· 12

Figure S17. HMBC spectrum of 2 ········· 13

Figure S18. NOESY spectrum of 2 ········· 13

Figure S19. ESIMS spectrum of 2 ········· 14

Figure S20. HRESIMS spectrum of 2 ········· 14

Figure S21. ^{1}H NMR spectrum of (600 MHz, $CDCl_3$) spectrum of 3 ········· 15

Figure S22. ^{13}C NMR spectrum of (150 MHz, $CDCl_3$) spectrum of 3 ········ 15

Figure S23. DEPT spectrum of 3 ········ 16

Figure S24. COSY spectrum of 3 ········ 16

Figure S25. HSQC spectrum of 3 ········ 17

Figure S26. HMBC spectrum of 3 ········ 17

Figure S27. NOESY spectrum of 3 ········ 18

Figure S28. EIMS spectrum of 1 ········ 19

Figure S29. ESIMS spectrum of 3 ········ 19

Figure S30. HRESIMS spectrum of 3 ········ 19

Figure S31. ^{1}H NMR spectrum of (600 MHz, $CDCl_3$) spectrum of 4 ········ 20

Figure S32. ^{13}C NMR spectrum of (150 MHz, $CDCl_3$) spectrum of 4 ········ 20

Figure S33. DEPT spectrum of 4 ········ 21

Figure S34. COSY spectrum of 4 ········ 21

Figure S35. HSQC spectrum of 4 ········ 22

Figure S36. HMBC spectrum of 4 ········ 22

Figure S37. NOESY spectrum of 4 ········ 23

Figure S38. ESIMS spectrum of 4 ········ 23

Figure S39. HRESIMS spectrum of 4 ········ 24

Figure S40. ^{1}H NMR spectrum of (400 MHz, $CDCl_3$) spectrum of 5 ········ 24

Figure S41. ^{13}C NMR spectrum of (100 MHz, $CDCl_3$) spectrum of 5 ········ 25

Figure S42. DEPT spectrum of 5 ········ 25

Figure S43. COSY spectrum of 5 ········ 26

Figure S44. **HSQC spectrum of 5** ···················· **26**

Figure S45. **HMBC spectrum of 5** ···················· **27**

Figure S46. **NOESY spectrum of 5** ···················· **27**

Figure S47. **ESIMS spectrum of 5** ···················· **28**

Figure S48. **HRESIMS spectrum of 5** ···················· **28**

Figure S49. **^{1}H NMR spectrum of (400 MHz, $CDCl_3$) spectrum of 6** ···················· **29**

Figure S50. **^{13}C NMR spectrum of (100 MHz, $CDCl_3$) spectrum of 6** ···················· **29**

Figure S51. **DEPT spectrum of 6** ···················· **30**

Figure S52. **COSY spectrum of 6** ···················· **30**

Figure S53. **HSQC spectrum of 6** ···················· **31**

Figure S54. **HMBC spectrum of 6** ···················· **31**

Figure S55. **HMBC spectrum of 6** ···················· **32**

Figure S56. **ESIMS spectrum of 6** ···················· **32**

Figure S57. **HRESIMS spectrum of 6** ···················· **33**

Figure S58. **Extraction and isolation of the leaves from *M. japonica* var. *kusanoi*** ···················· **33**

Figure S59. **^{1}H NMR spectrum of (600 MHz, $CDCl_3$) spectrum of 4a** ···················· **34**

Figure S60. **^{1}H NMR spectrum of (600 MHz, $CDCl_3$) spectrum of 4b** ···················· **34**

Figure S61. **^{1}H NMR spectrum of (600 MHz, $CDCl_3$) spectrum of 5a** ···················· **35**

Figure S62. **^{1}H NMR spectrum of (600 MHz, $CDCl_3$) spectrum of 5b** ···················· **35**

Figure S63. **^{1}H NMR spectrum of (400 MHz, $CDCl_3$) spectrum of 6a** ···················· **36**

Figure S64. **^{1}H NMR spectrum of (400 MHz, $CDCl_3$) spectrum of 6b** ···················· **36**

Table S1. Inhibitory effects of crude extracts from the leaves of *M. japonica* var. *kusanoi* on superoxide anion generation and elastase release in fMLP/CB-induced human neutrophils. 37

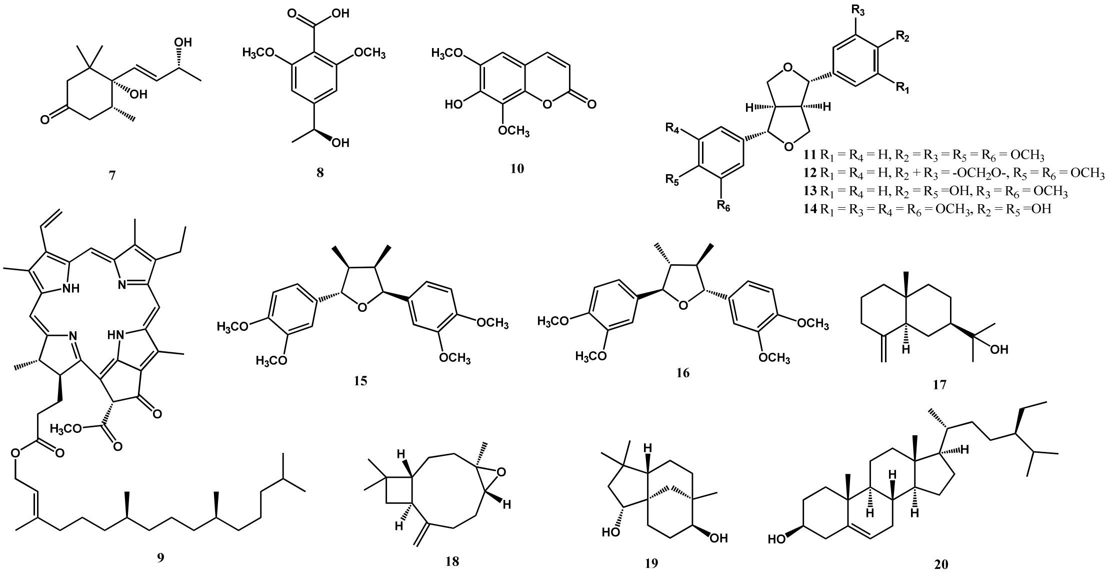

Figure S1. Structures of known compounds **7–20**.

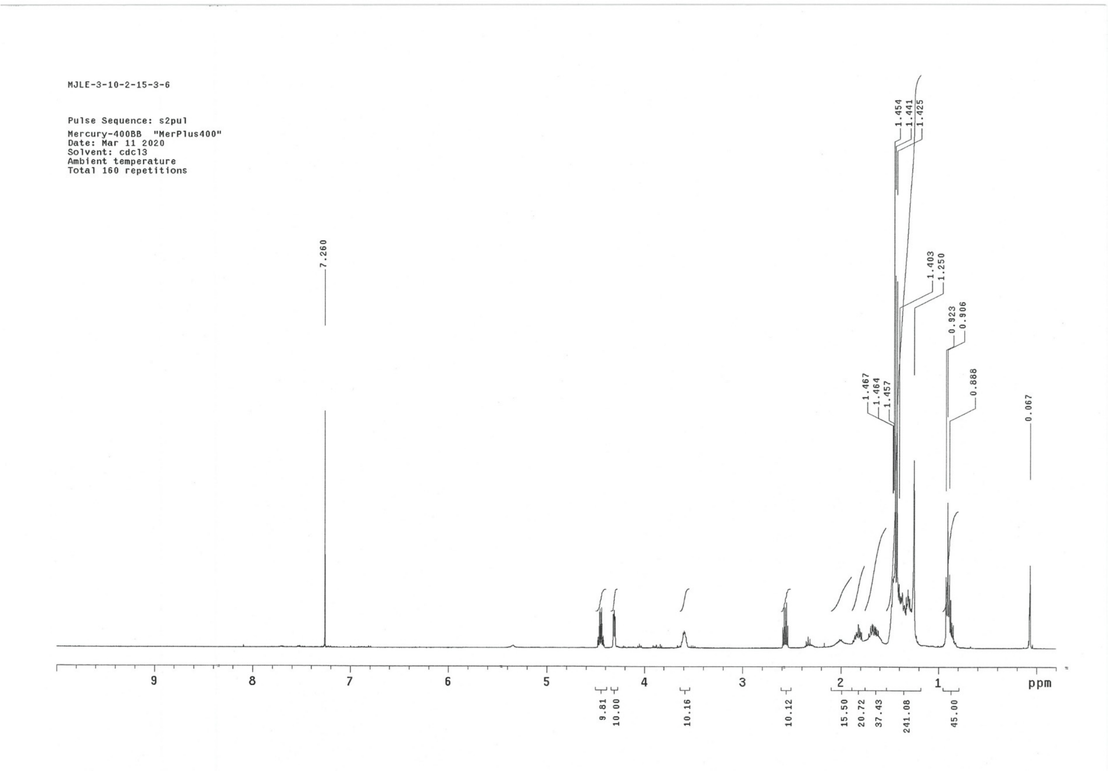

Figure S2. ^{1}H NMR spectrum of (400 MHz, $CDCl_3$) spectrum of **1**

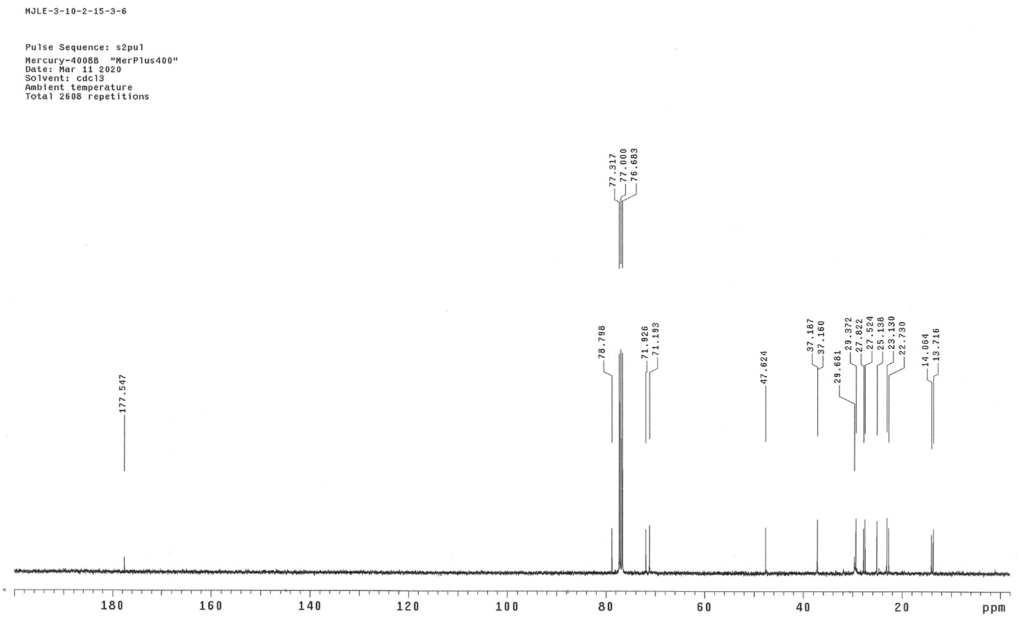

Figure S3. ^{13}C NMR spectrum of (100 MHz, $CDCl_3$) spectrum of **1**

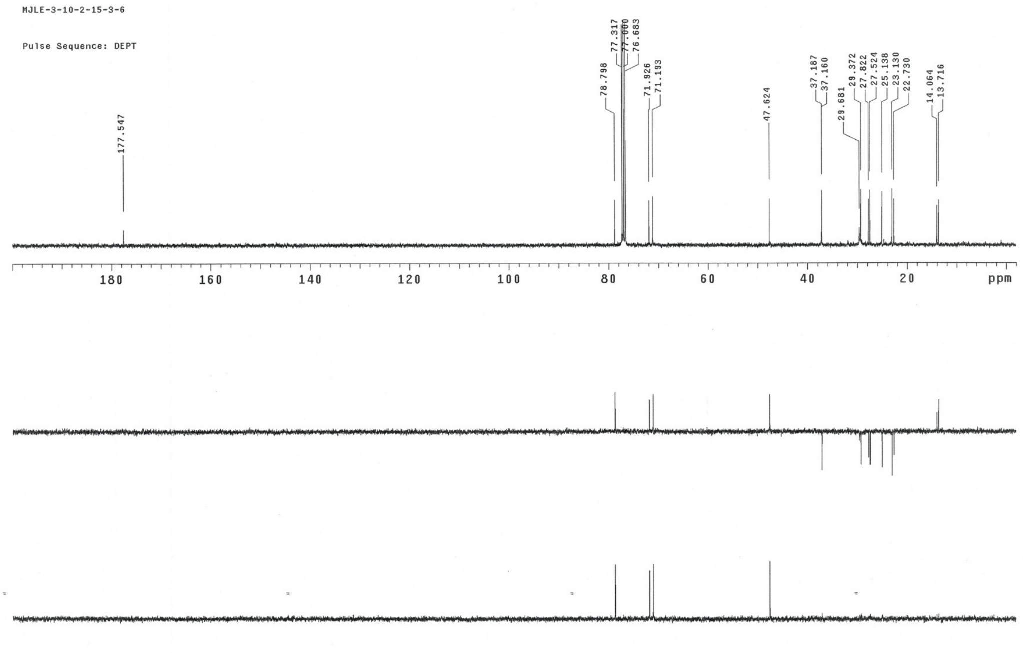

Figure S4. DEPT spectrum of **1**

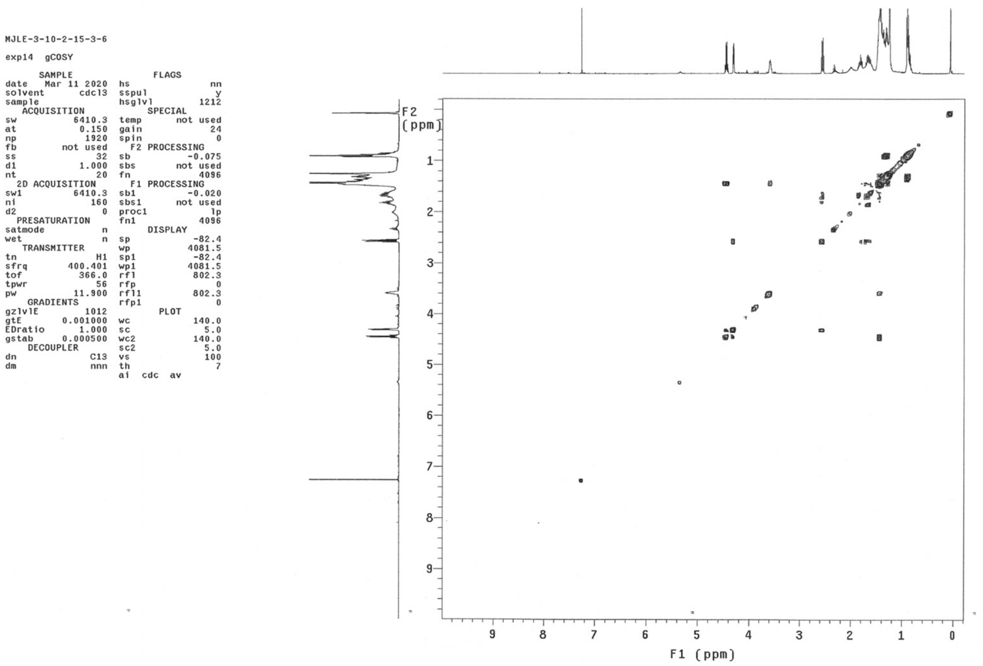

Figure S5. COSY spectrum of **1**

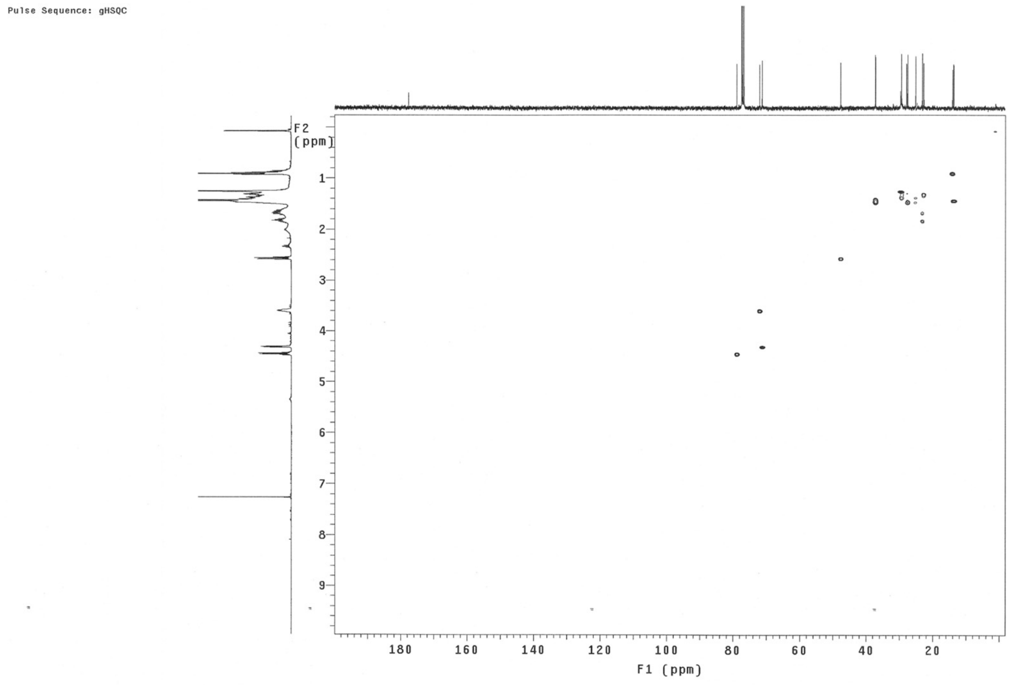

Figure S6. HSQC spectrum of **1**

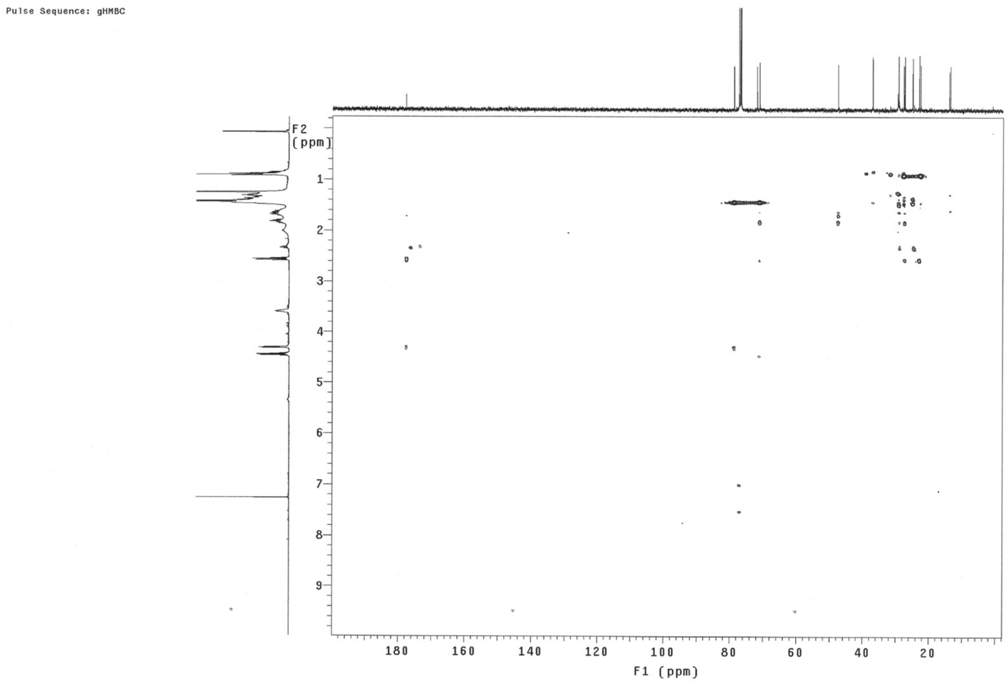

Figure S7. HMBC spectrum of **1**

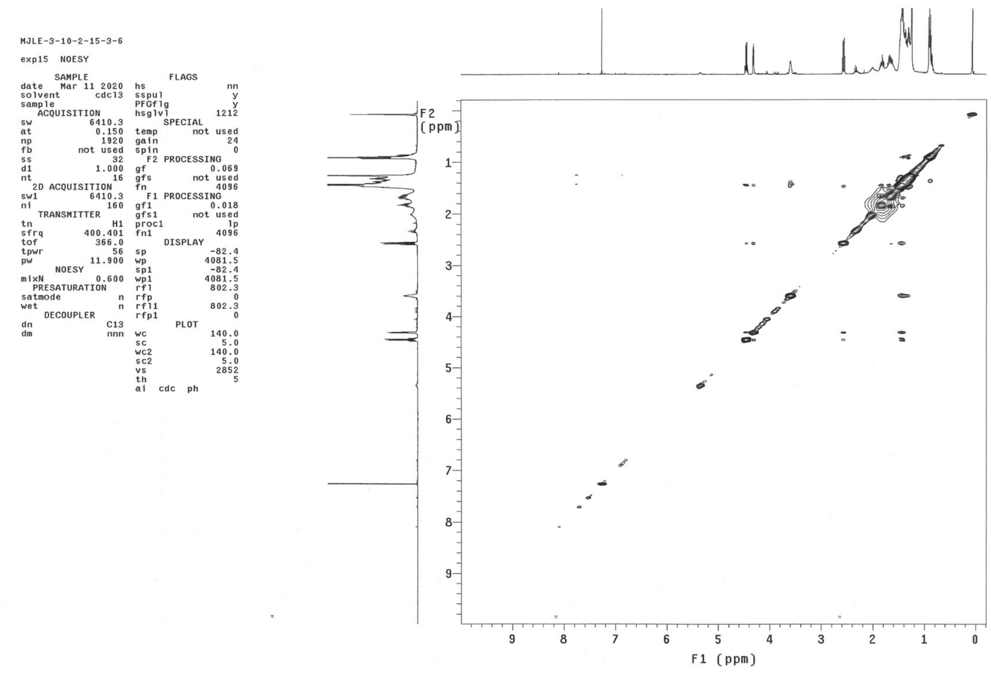

Figure S8. NOESY spectrum of **1**

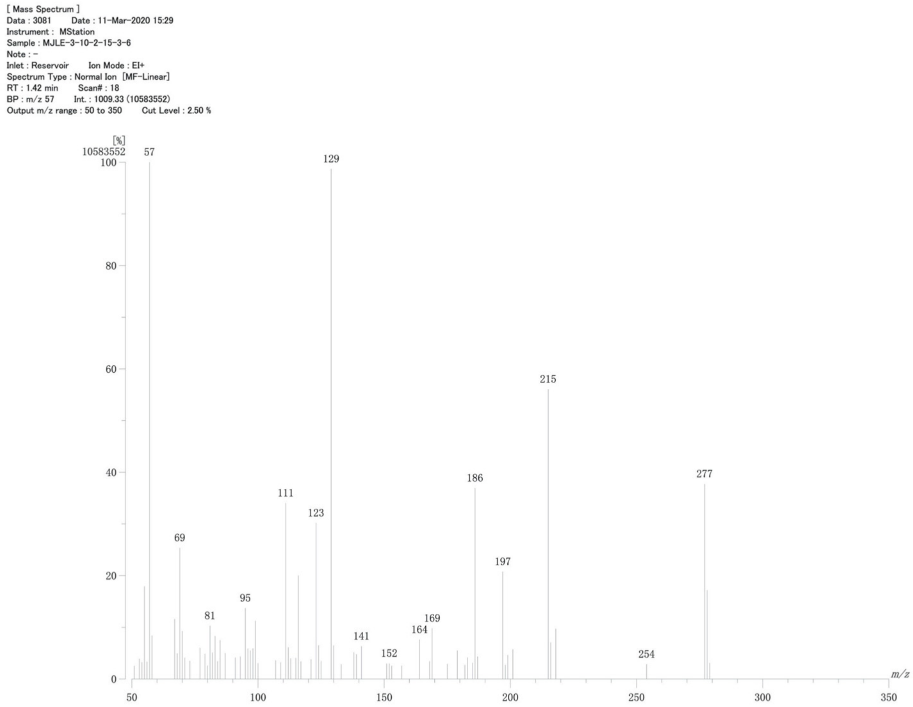

Figure S9. EIMS spectrum of **1**

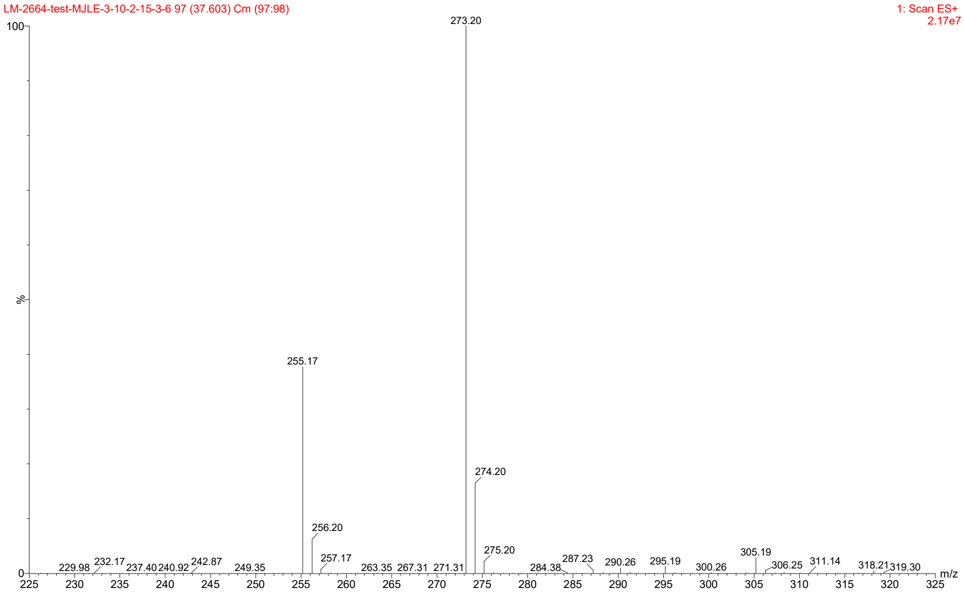

Figure S10. ESIMS spectrum of **1**

Data:MJLE-3-10-2-15-3-6
Comment:
Description:
Ionization Mode:ESI+
History:Average(MS[1] 0.37..0.38)

Acquired:2/7/2020 1:58:58 PM
Operator:AccuTOF
m/z Calibration File:20200102-1TFANa...
Created:2/7/2020 2:02:24 PM
Created by:AccuTOF

Charge number:1 Tolerance:250.00[ppm], 200.00 .. 200.... Unsaturation Number:-100.5 .. 200.0 (...
Element:^{12}C:15 .. 15, ^{1}H:28 .. 29, ^{23}Na:0 .. 2, ^{16}O:4 .. 4

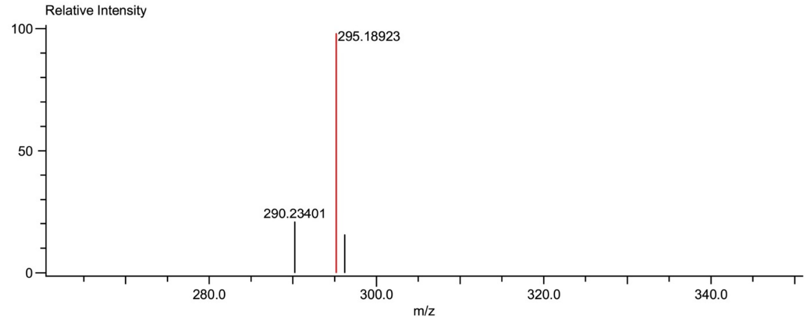

Mass	Intensity	Calc. Mass	Mass Difference [mDa]	Mass Difference [ppm]	Possible Formula
295.18923	13277.94	295.18853	0.70	2.36	$^{12}C_{15}{}^{1}H_{28}{}^{23}Na_{1}{}^{16}O_{4}$

Figure S11. HRESIMS spectrum of **1**

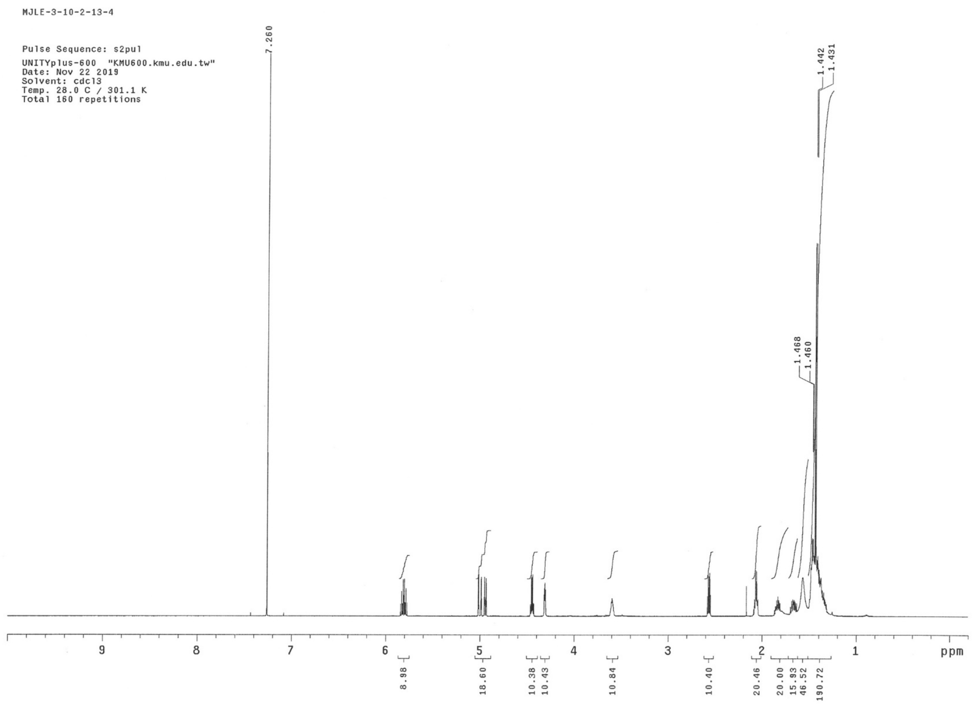

Figure S12. ^{1}H NMR spectrum of (600 MHz, $CDCl_3$) spectrum of **2**

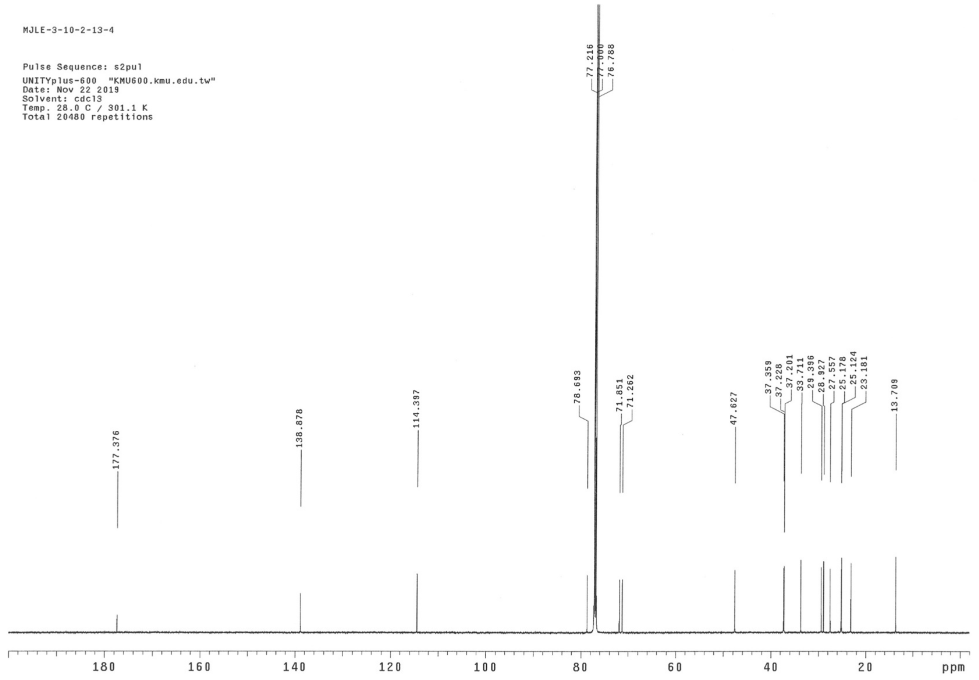

Figure S13. ¹³C NMR spectrum of (150 MHz, $CDCl_3$) spectrum of **2**

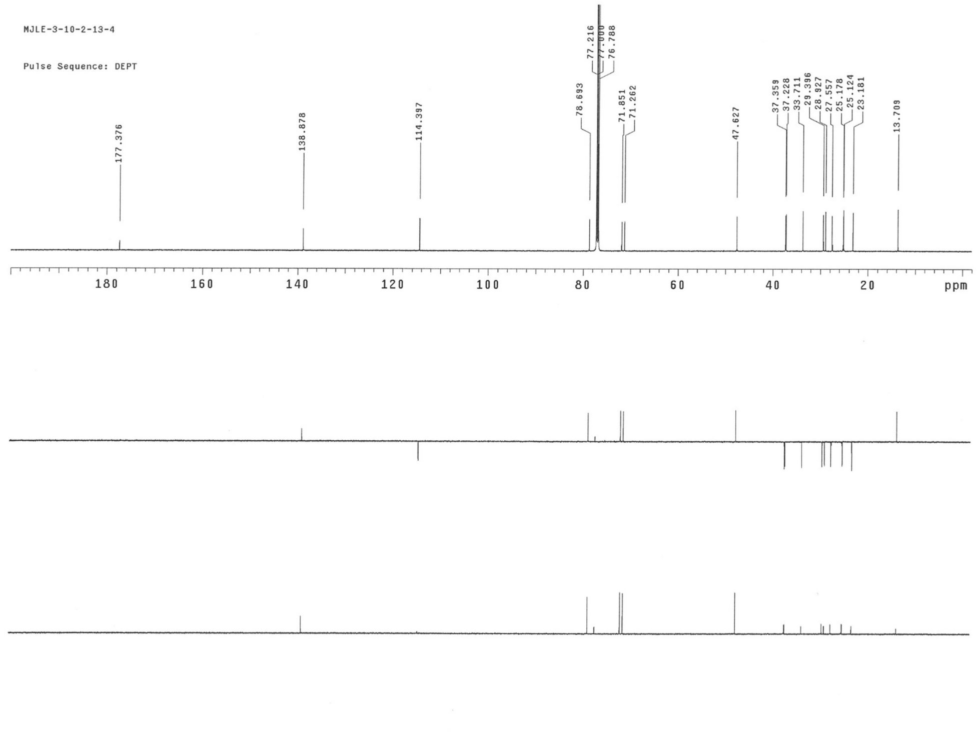

Figure S14. DEPT spectrum of **2**

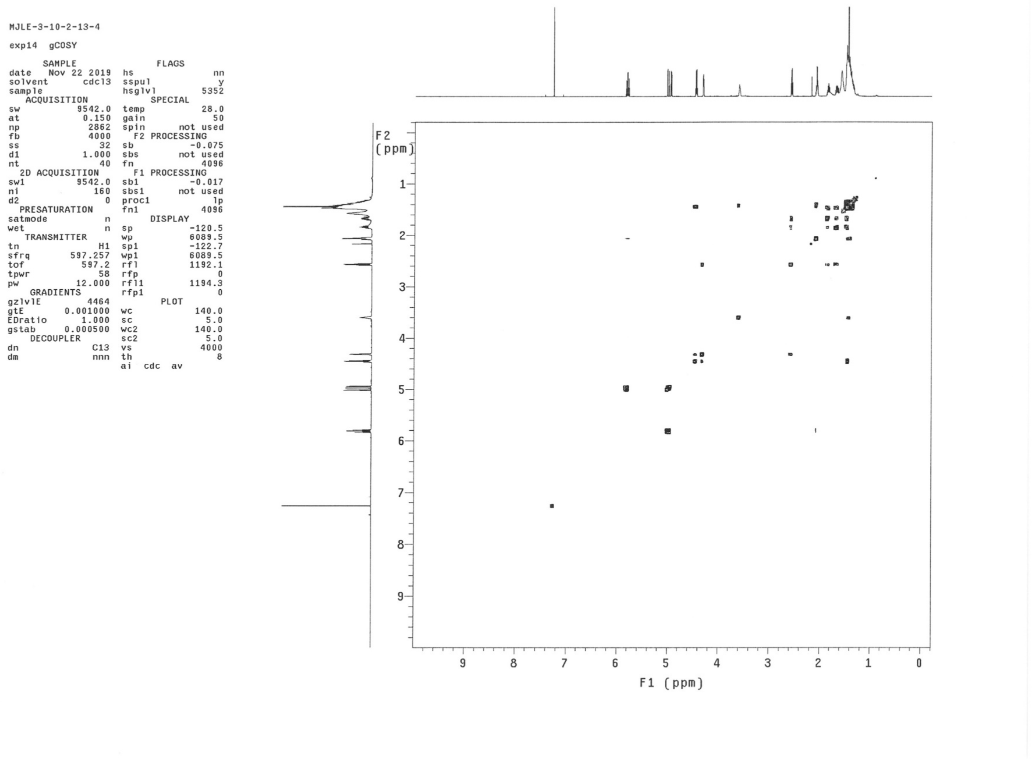

Figure S15. COSY spectrum of **2**

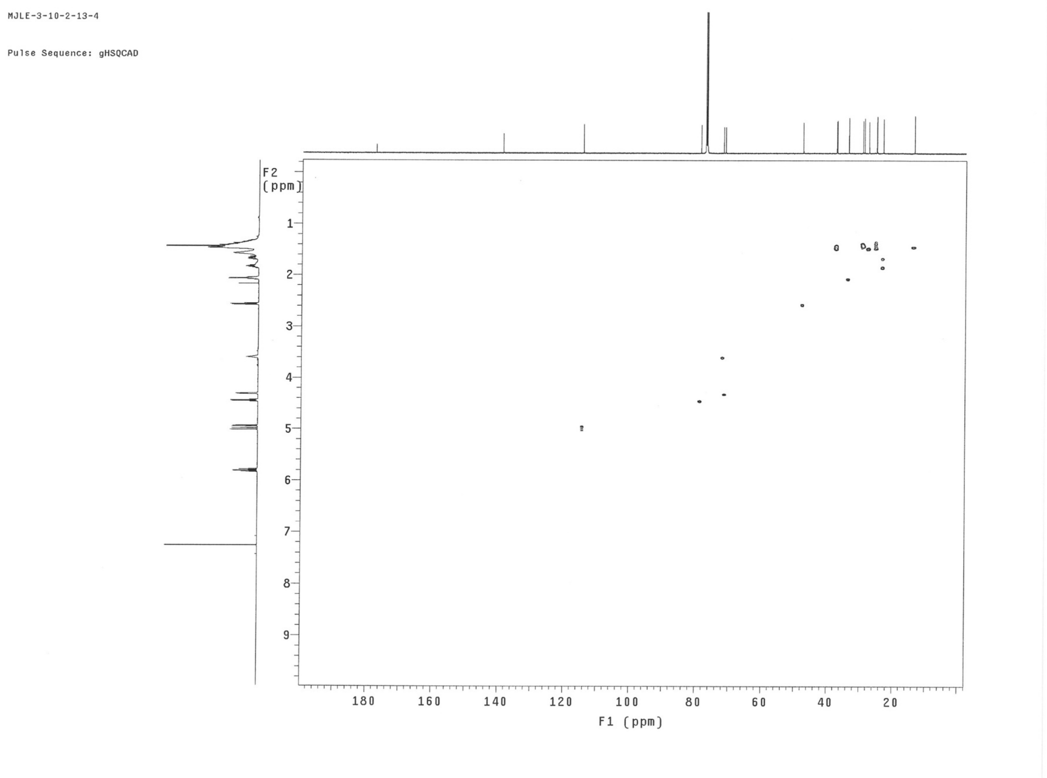

Figure S16. HSQC spectrum of **2**

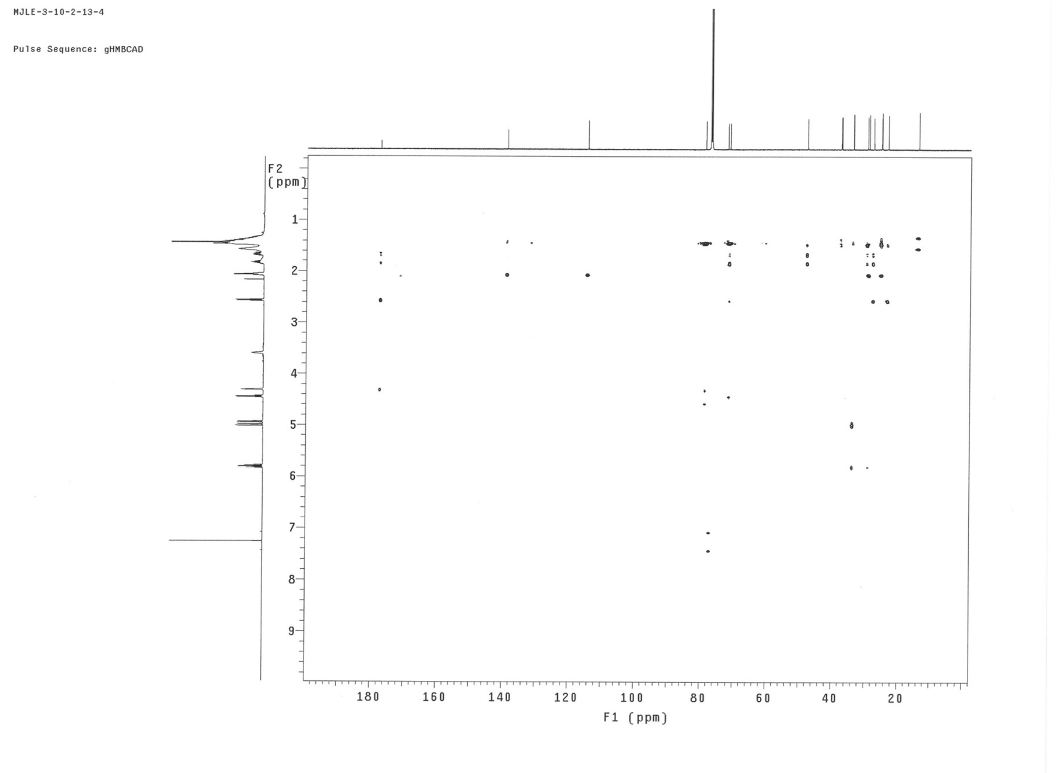

Figure S17. HMBC spectrum of **2**

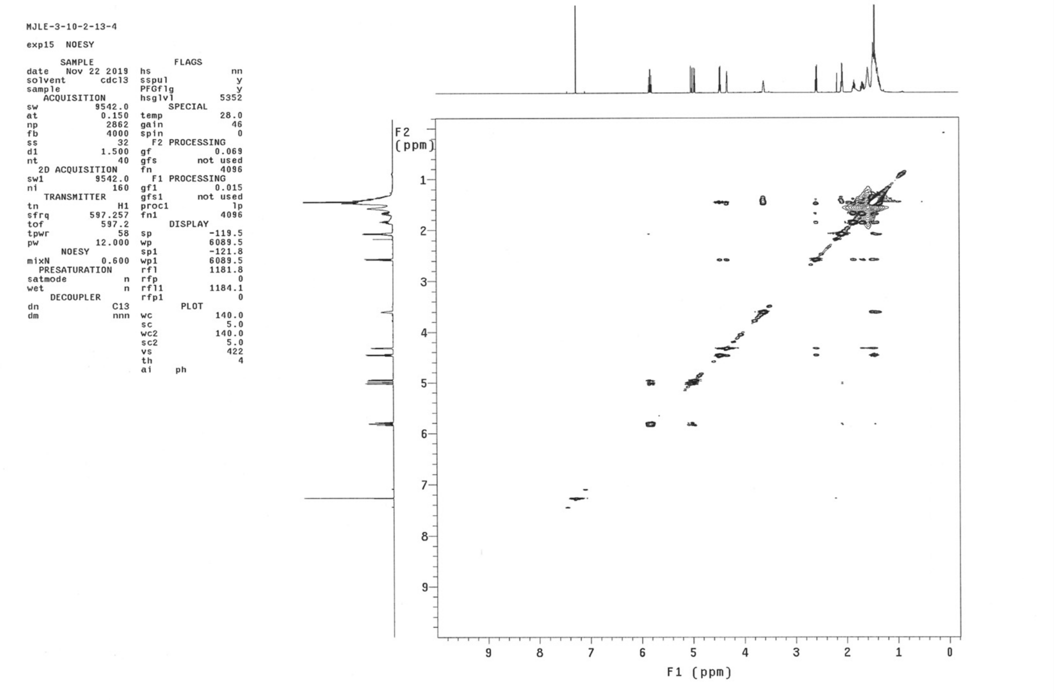

Figure S18. NOESY spectrum of **2**

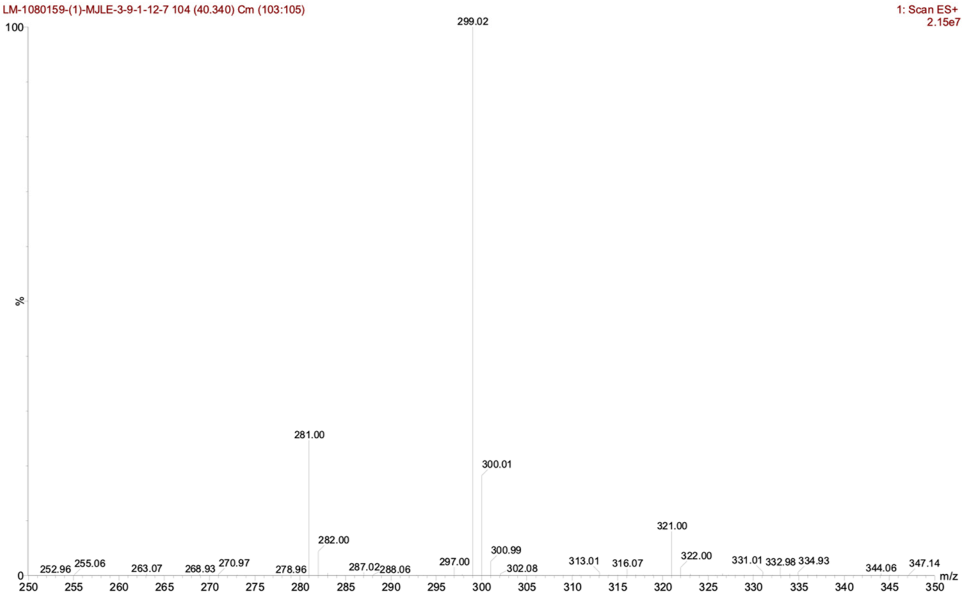

Figure S19. ESIMS spectrum of **2**

Mass Spectrum SmartFormula Report

Analysis Info

Analysis Name	D:\Data\f7\MJLE3102134_000005.d	12/31/2019 3:14:11 PM
Method	broadband first signal	Operator: YU HSIAO-CHING
Sample Name	MJLE-3-10-2-13-4	Instrument: BRUKER FT-MS solariX
Comment	ESI Positive	

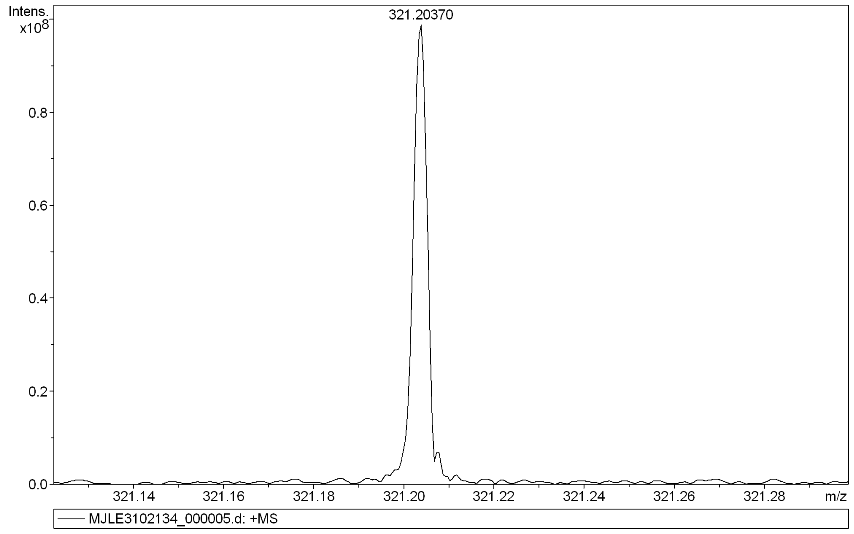

Meas. m/z	#	Formula	Score	m/z	err [mDa]	err [ppm]	mSigma	rdb	e⁻ Conf	N-Rule
321.20370	1	C 17 H 30 Na O 4	100.00	321.20363	-0.07	-0.23	18.8	2.5	even	ok

Figure S20. HRESIMS spectrum of **2**

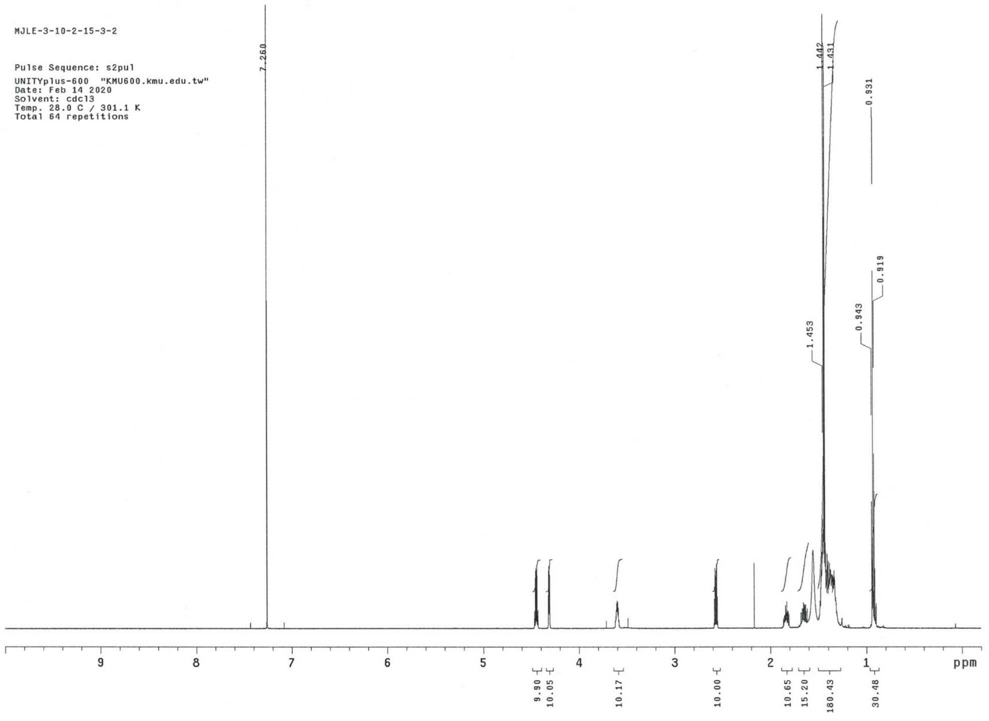

Figure S21. ^{1}H NMR spectrum of (600 MHz, $CDCl_3$) spectrum of **3**

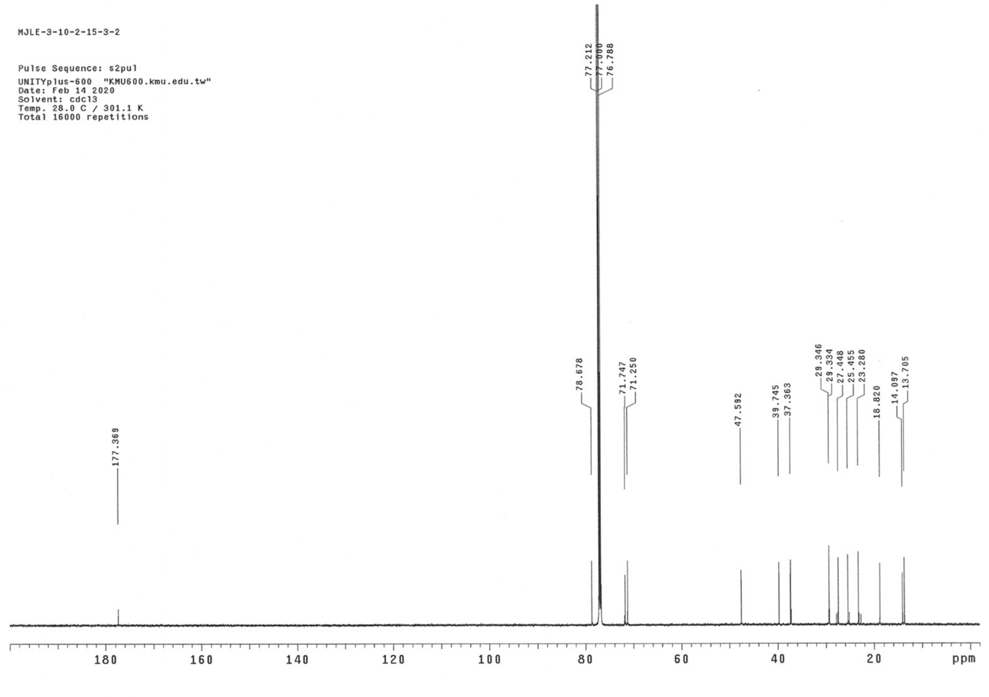

Figure S22. ^{13}C NMR spectrum of (150 MHz, $CDCl_3$) spectrum of **3**

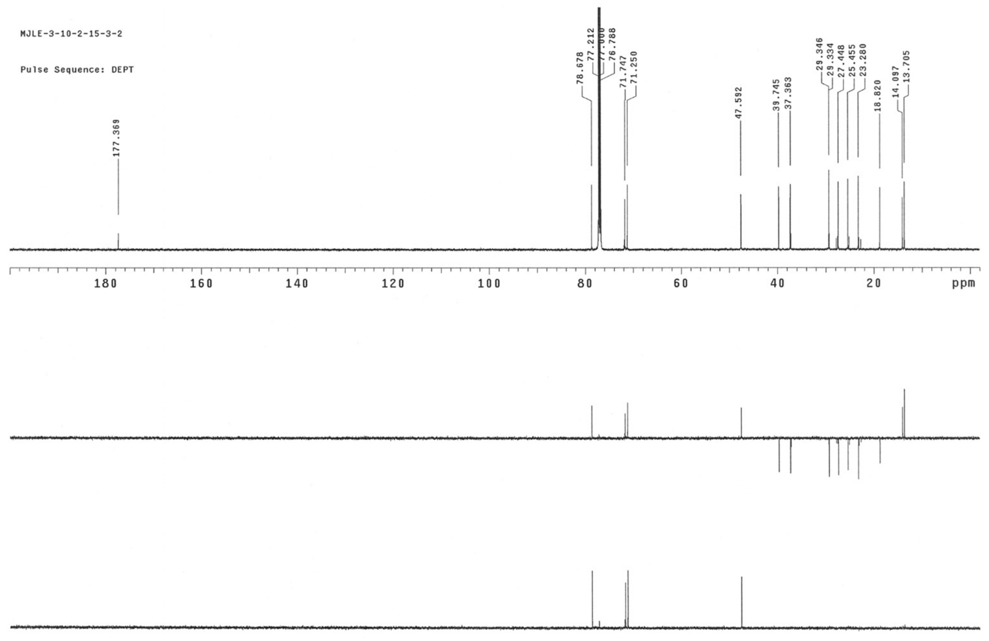

Figure S23. DEPT spectrum of **3**

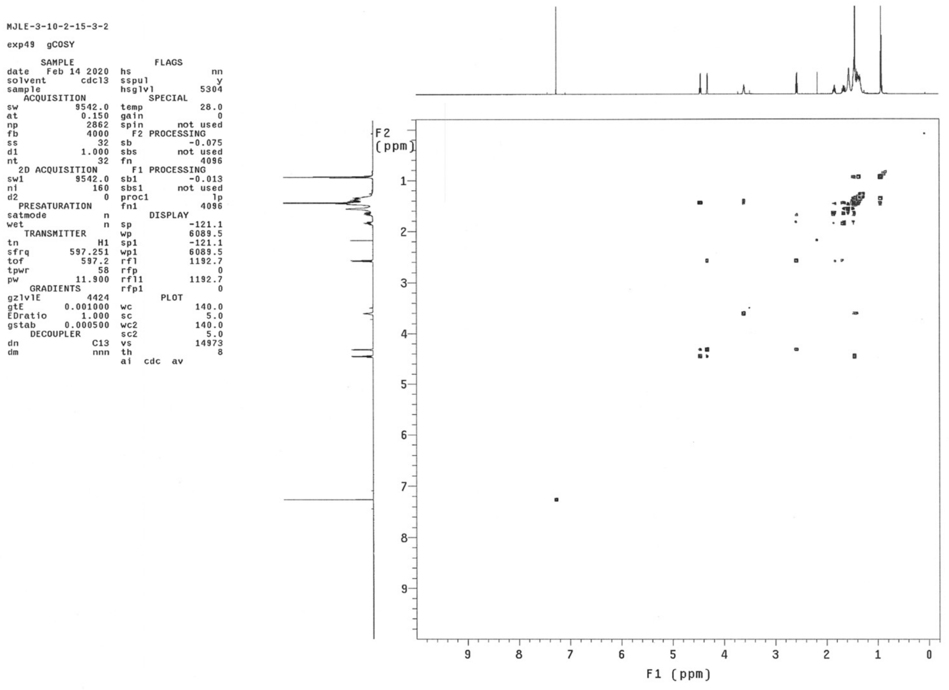

Figure S24. COSY spectrum of **3**

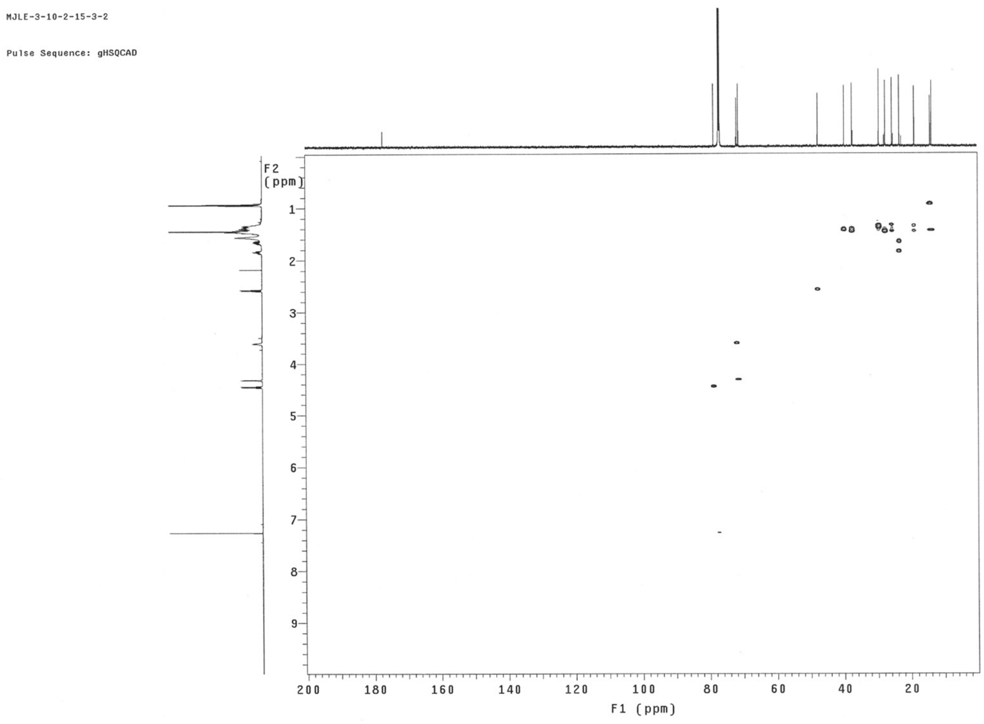

Figure S25. HSQC spectrum of **3**

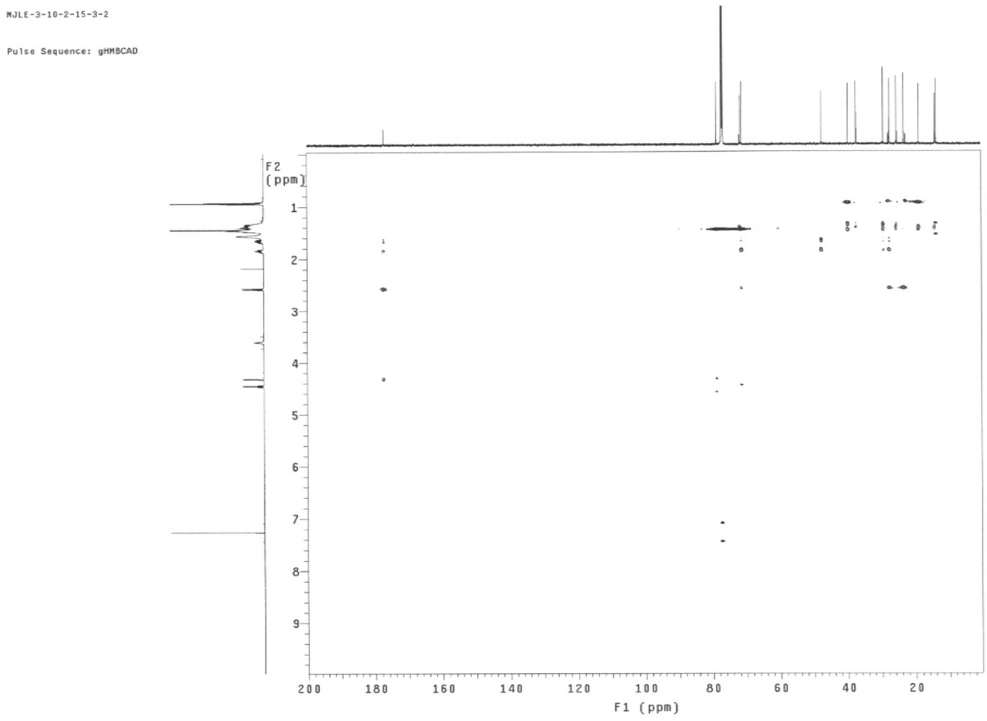

Figure S26. HMBC spectrum of **3**

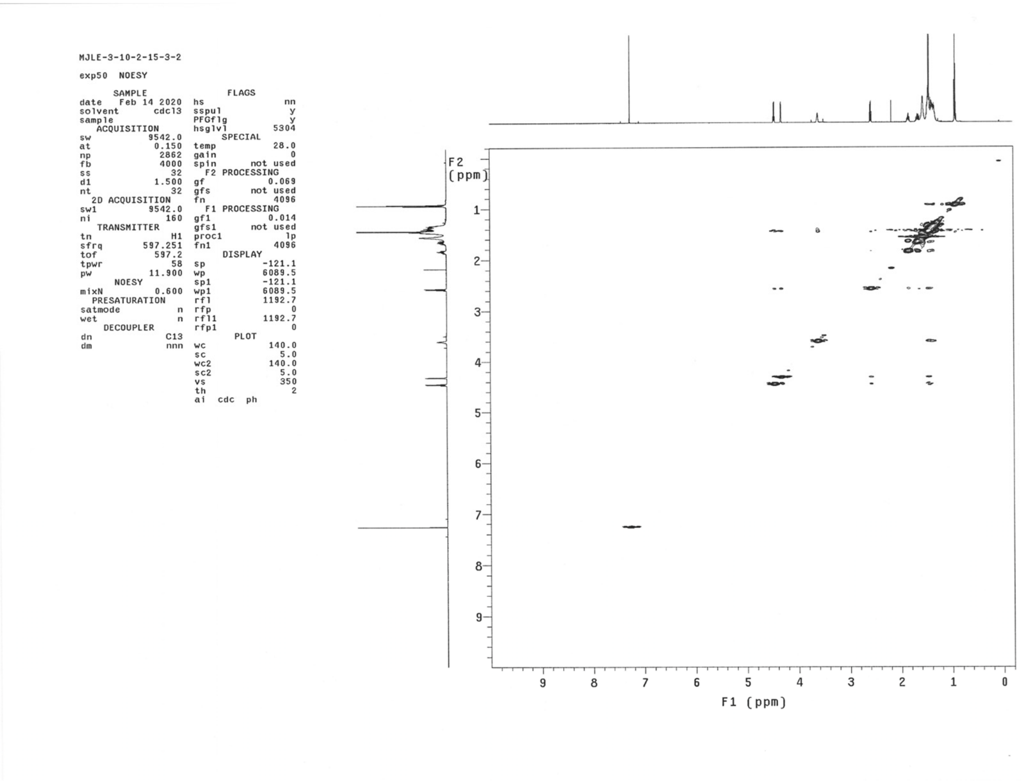

Figure S27. NOESY spectrum of **3**

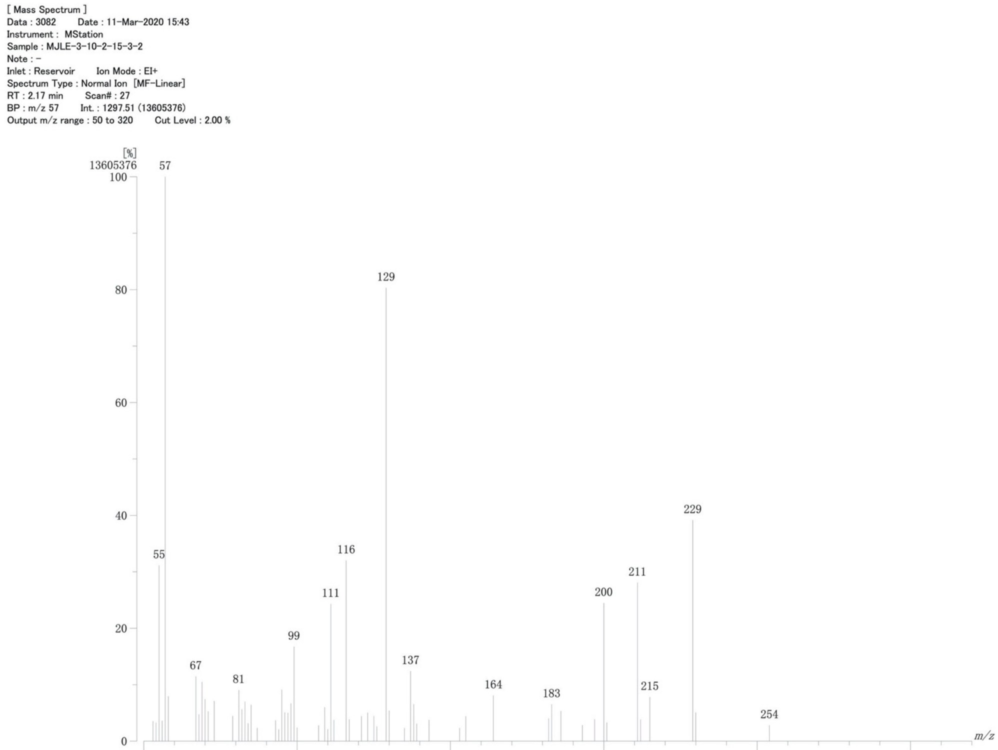

Figure S28. EIMS spectrum of **1**

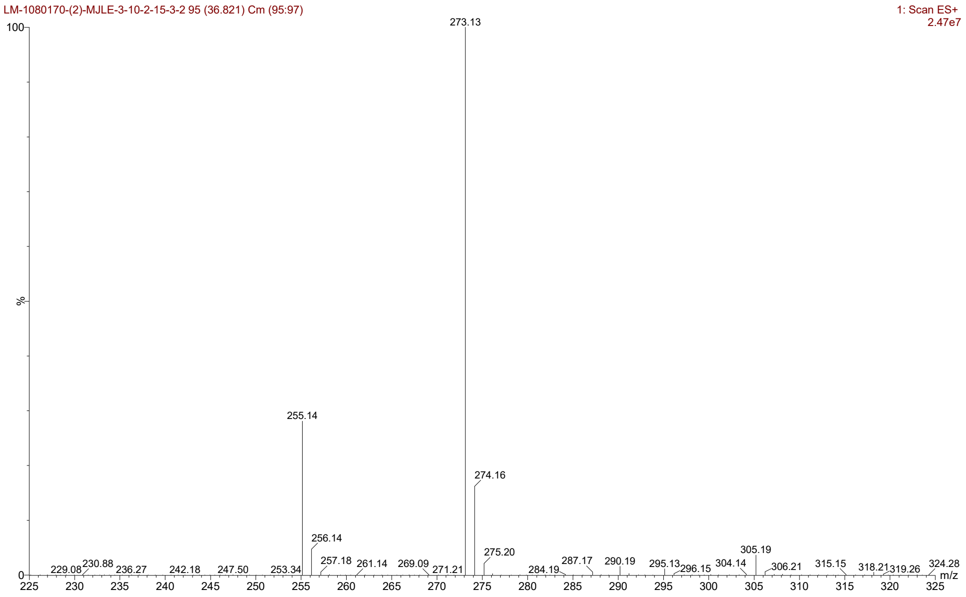

Figure S29. ESIMS spectrum of **3**

Data:MJLE-3-10-2-15-3-2
Comment:
Description:
Ionization Mode:ESI+
History:Average(MS[1] 0.38..0.39)

Acquired:12:00:00 AM
Operator:AccuTOF
m/z Calibration File:20200102-1TFANa...
Created:2/7/2020 2:00:40 PM
Created by:AccuTOF

Charge number:1 Tolerance:250.00[ppm], 200.00 .. 200.... Unsaturation Number:-100.5 .. 200.0 (...
Element:^{12}C:15 .. 15, ^{1}H:28 .. 29, ^{23}Na:0 .. 2, ^{16}O:4 .. 4

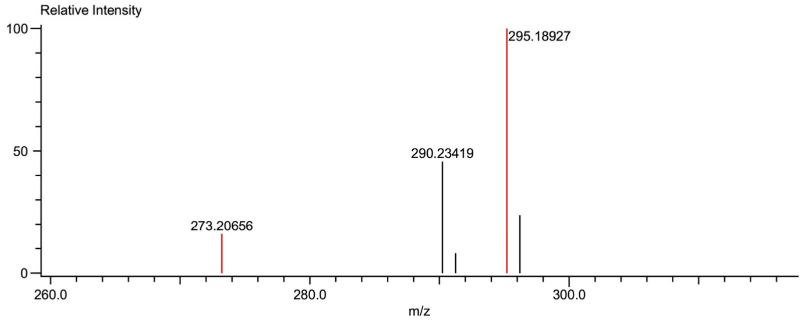

Mass	Intensity	Calc. Mass	Mass Difference [mDa]	Mass Difference [ppm]	Possible Formula
273.20656	1360.64	273.20658	-0.02	-0.07	$^{12}C_{15}{}^{1}H_{29}{}^{16}O_4$
295.18927	8664.71	295.18853	0.74	2.52	$^{12}C_{15}{}^{1}H_{28}{}^{23}Na_1{}^{16}O_4$

Figure S30. HRESIMS spectrum of **3**

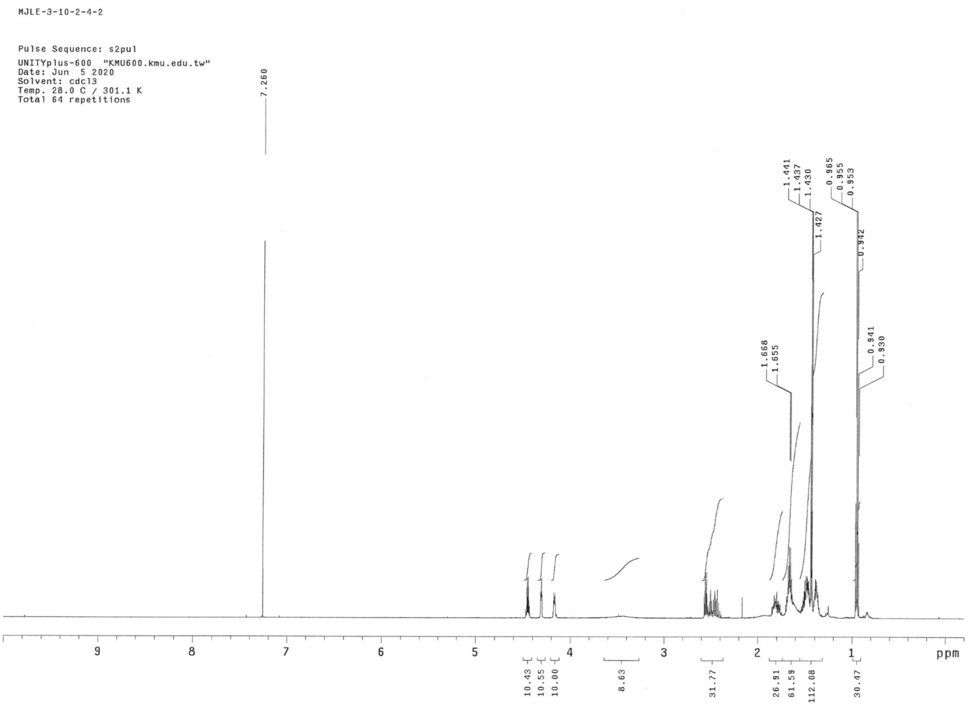

Figure S31. ^{1}H NMR spectrum of (600 MHz, $CDCl_3$) spectrum of **4**

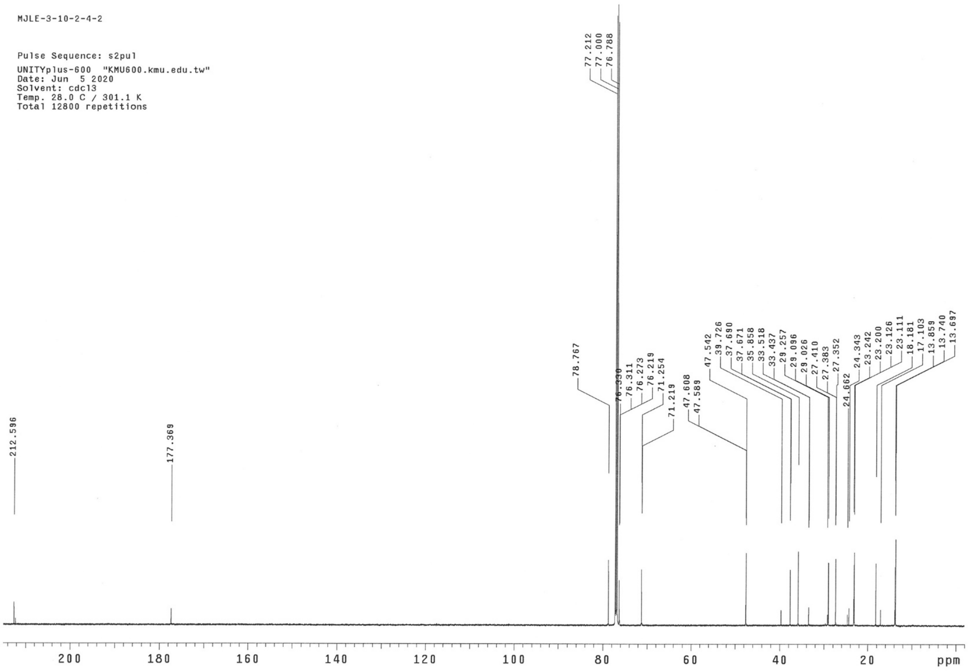

Figure S32. ^{13}C NMR spectrum of (150 MHz, $CDCl_3$) spectrum of **4**

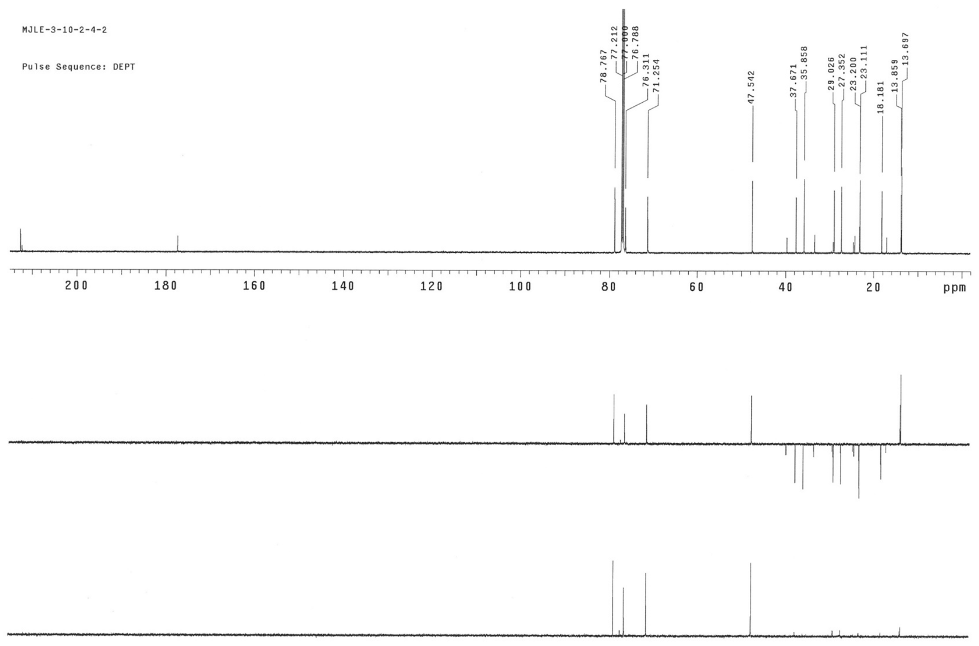

Figure S33. DEPT spectrum of **4**

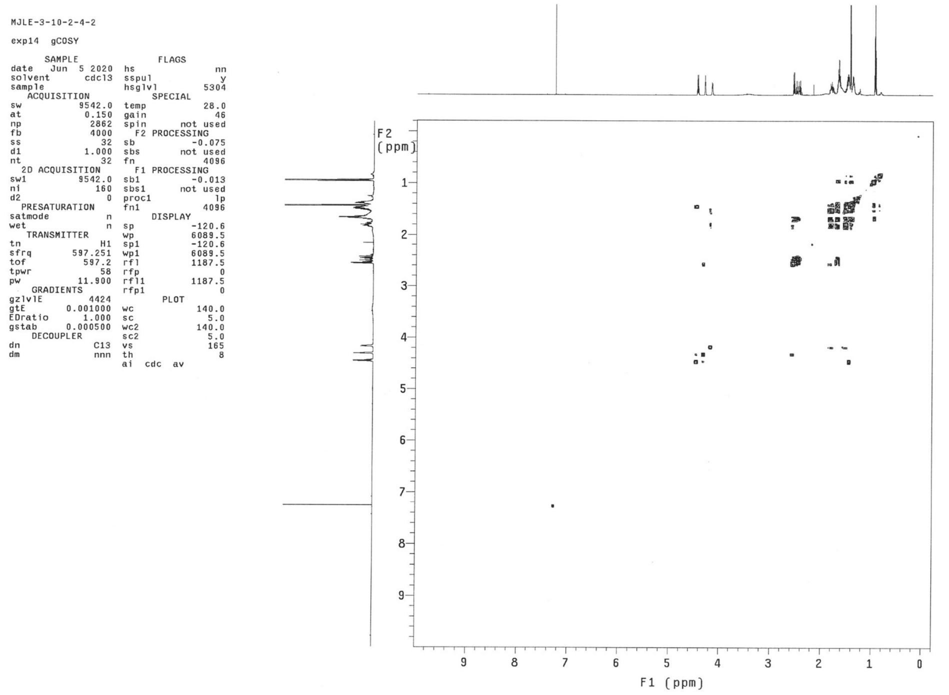

Figure S34. COSY spectrum of **4**

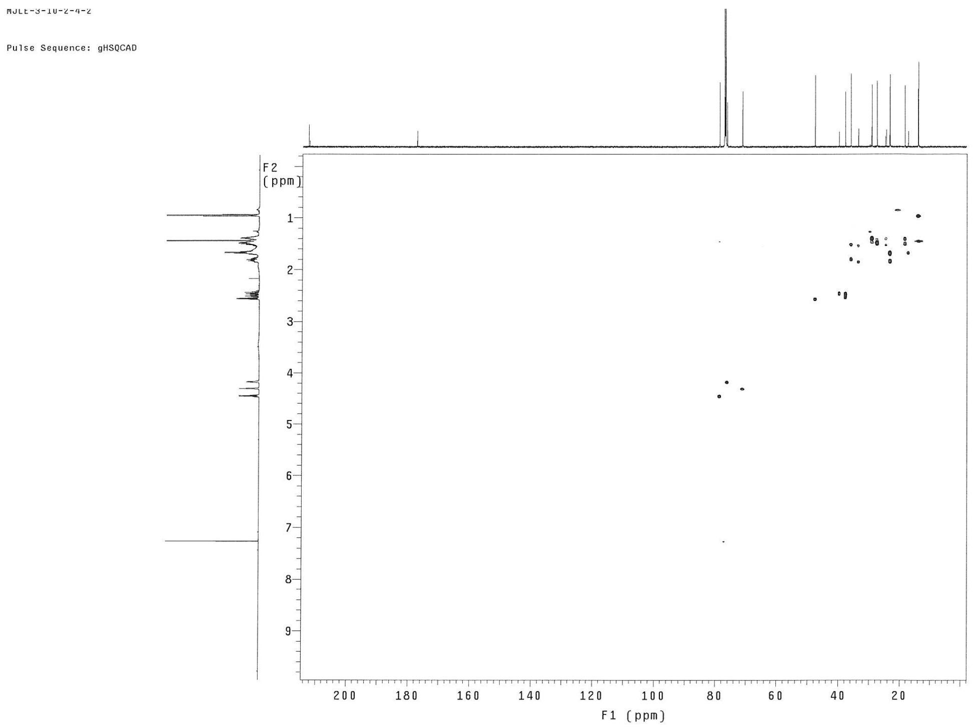

Figure S35. HSQC spectrum of **4**

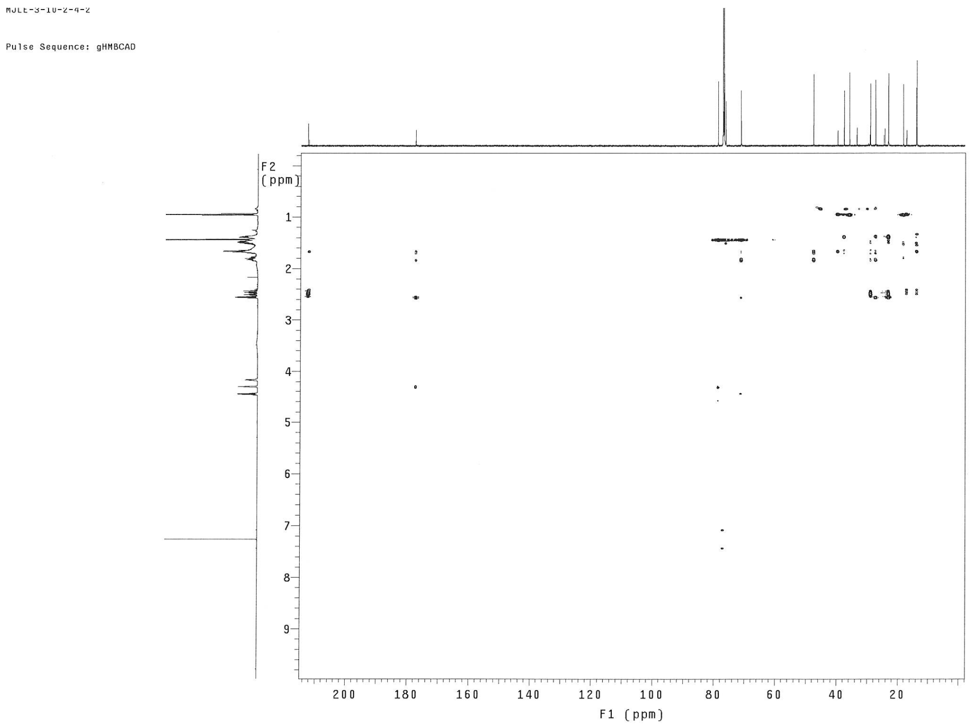

Figure S36. HMBC spectrum of **4**

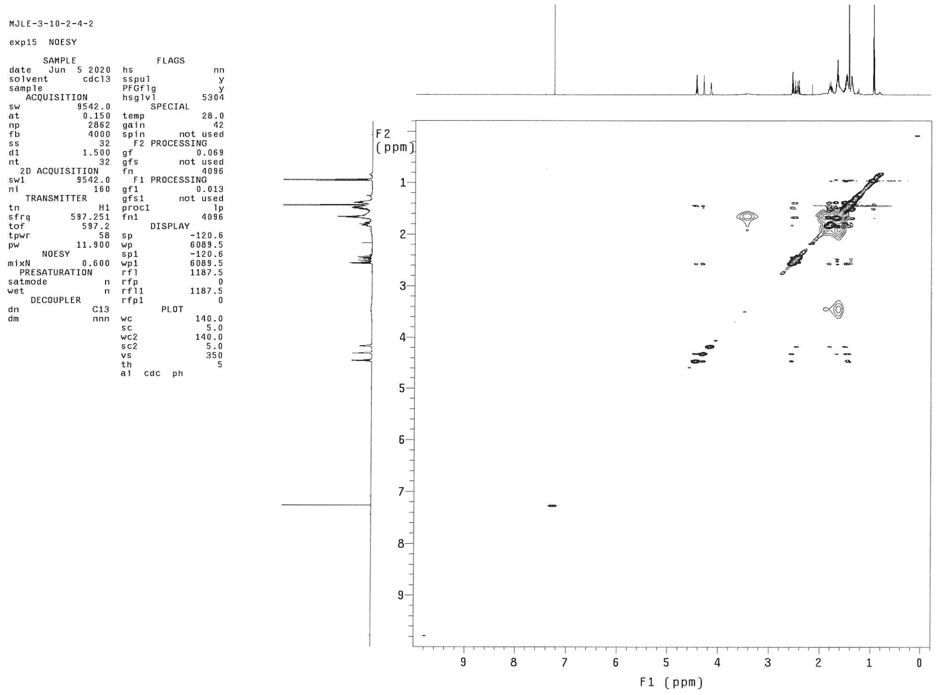

Figure S37. NOESY spectrum of **4**

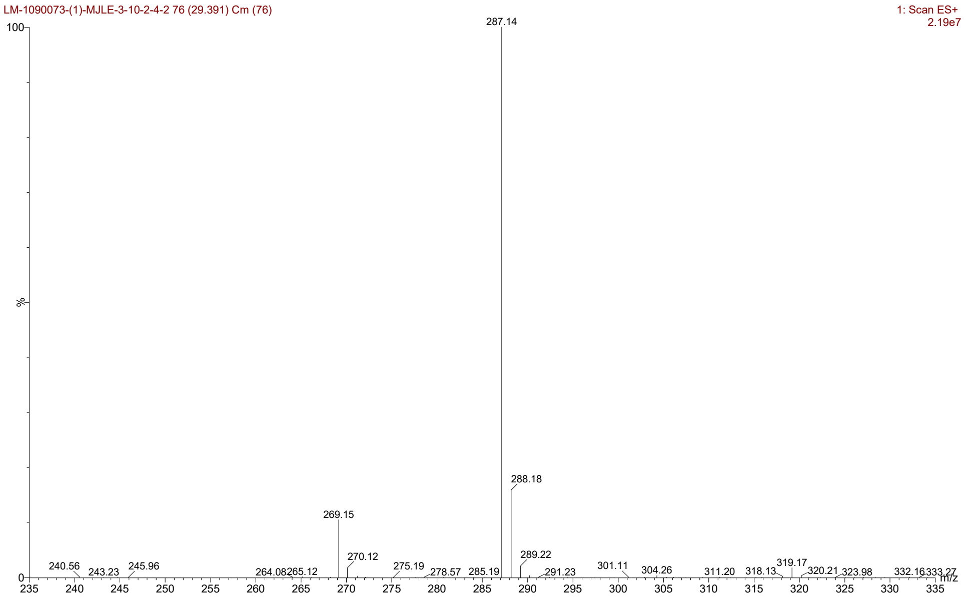

Figure S38. ESIMS spectrum of **4**

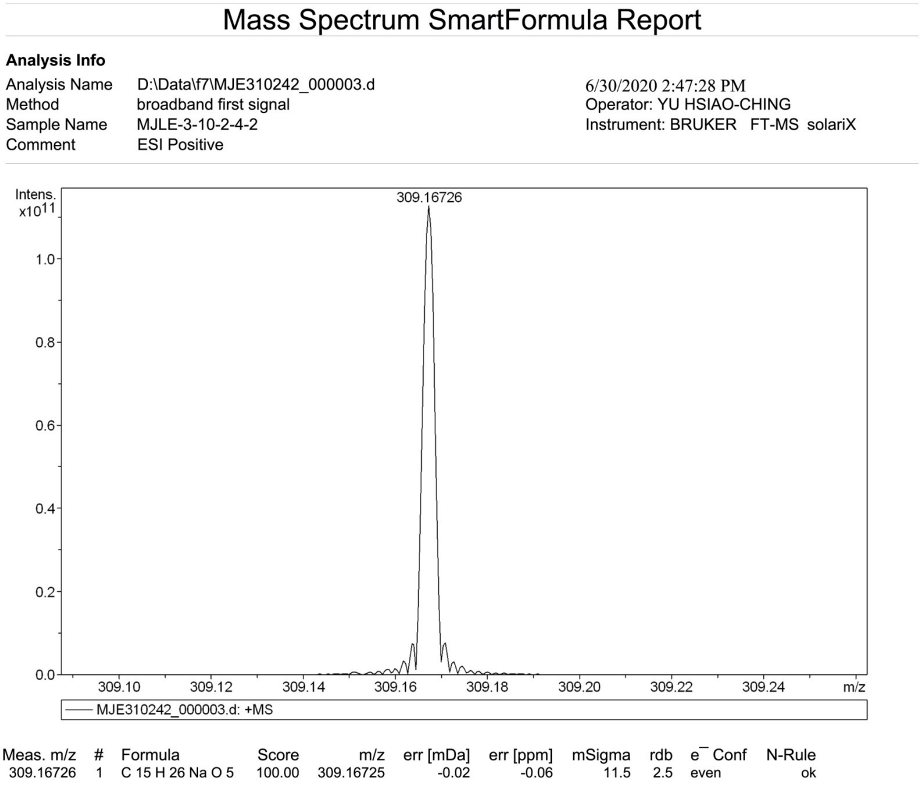

Figure S39. HRESIMS spectrum of **4**

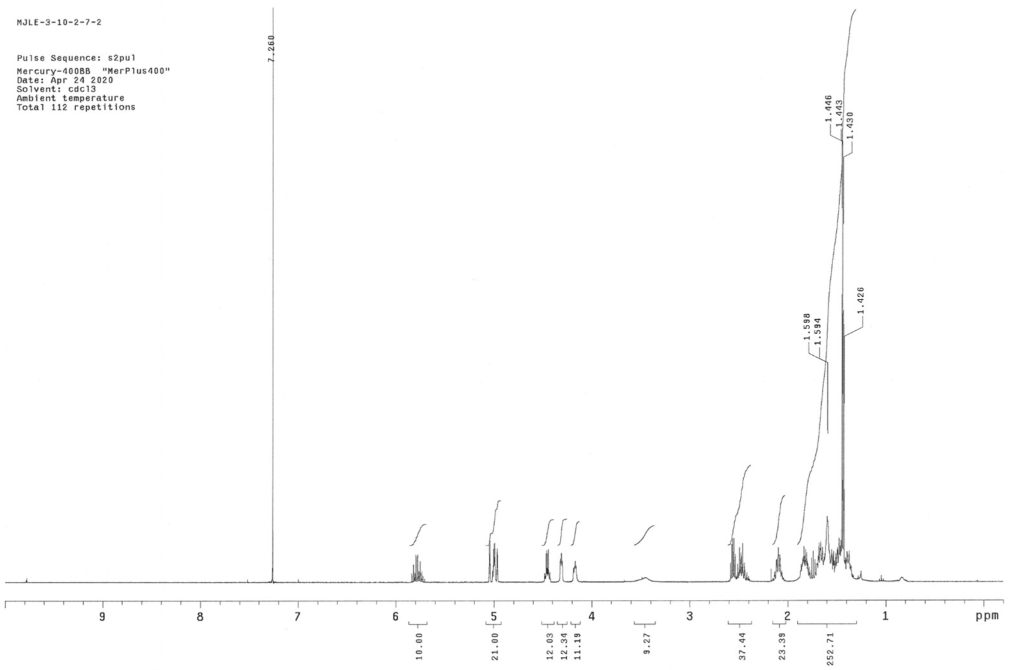

Figure S40. ^{1}H NMR spectrum of (400 MHz, $CDCl_3$) spectrum of **5**

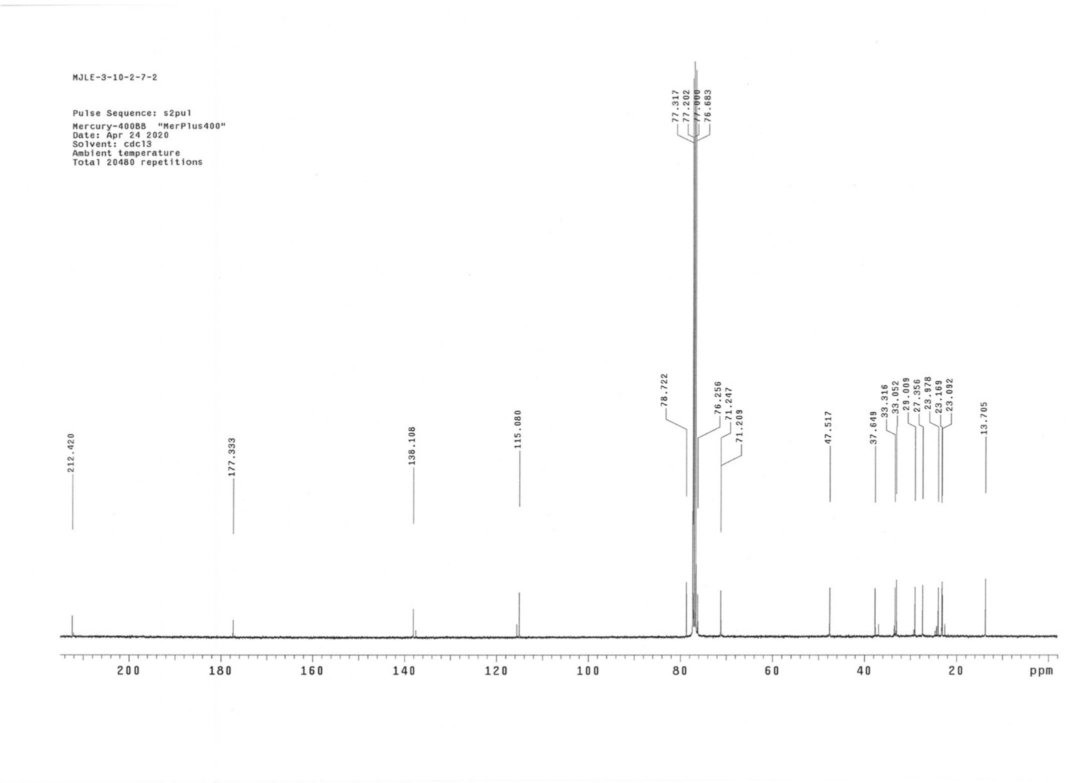

Figure S41. ^{13}C NMR spectrum of (100 MHz, $CDCl_3$) spectrum of **5**

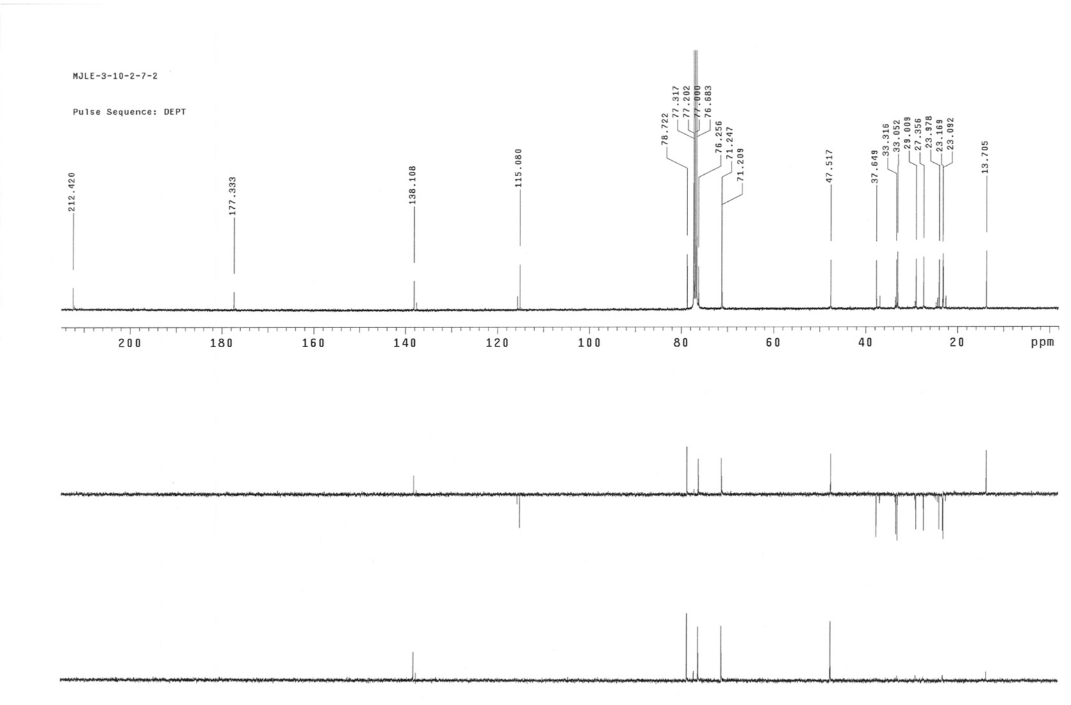

Figure S42. DEPT spectrum of **5**

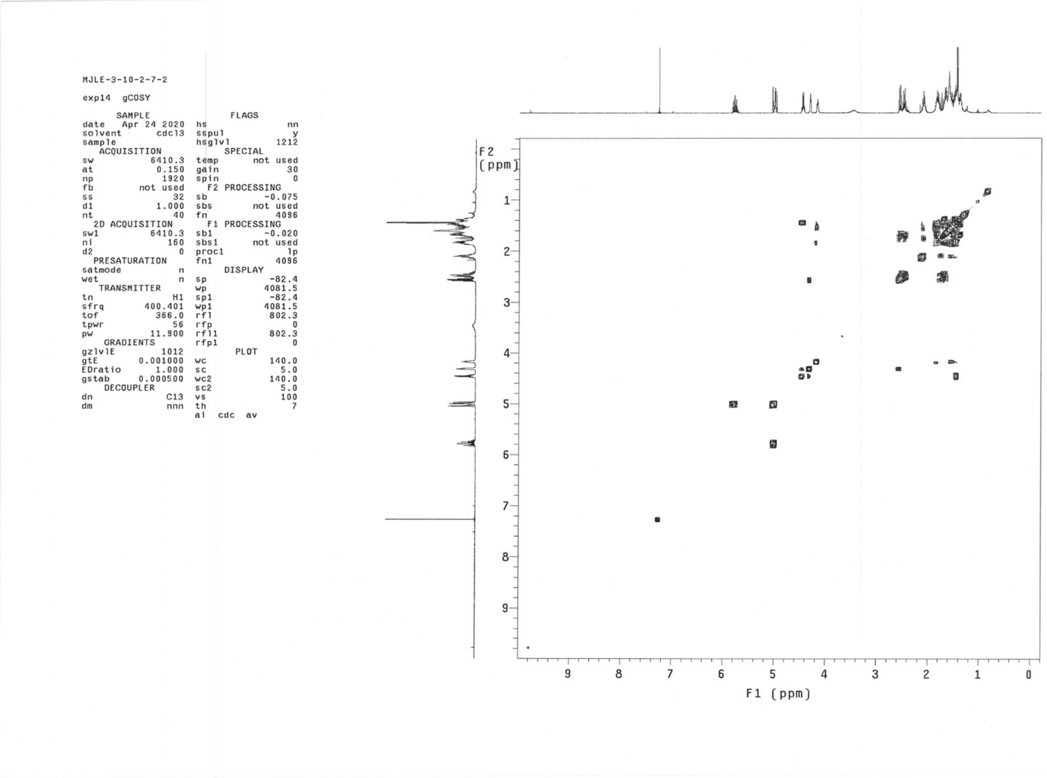

Figure S43. COSY spectrum of **5**

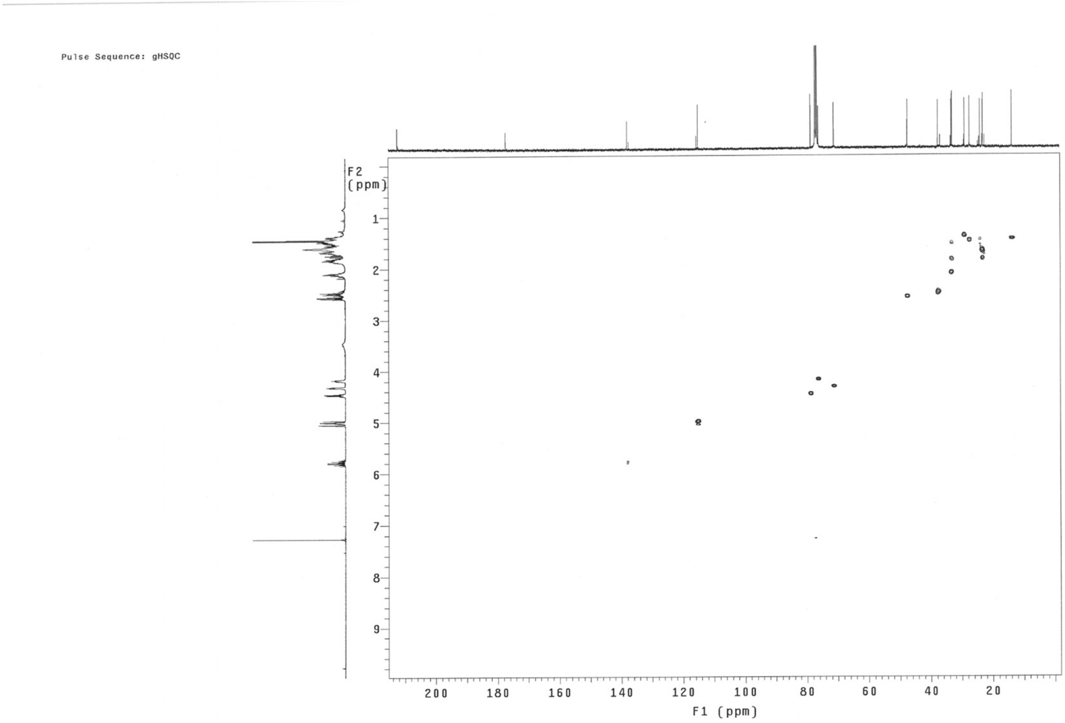

Figure S44. HSQC spectrum of **5**

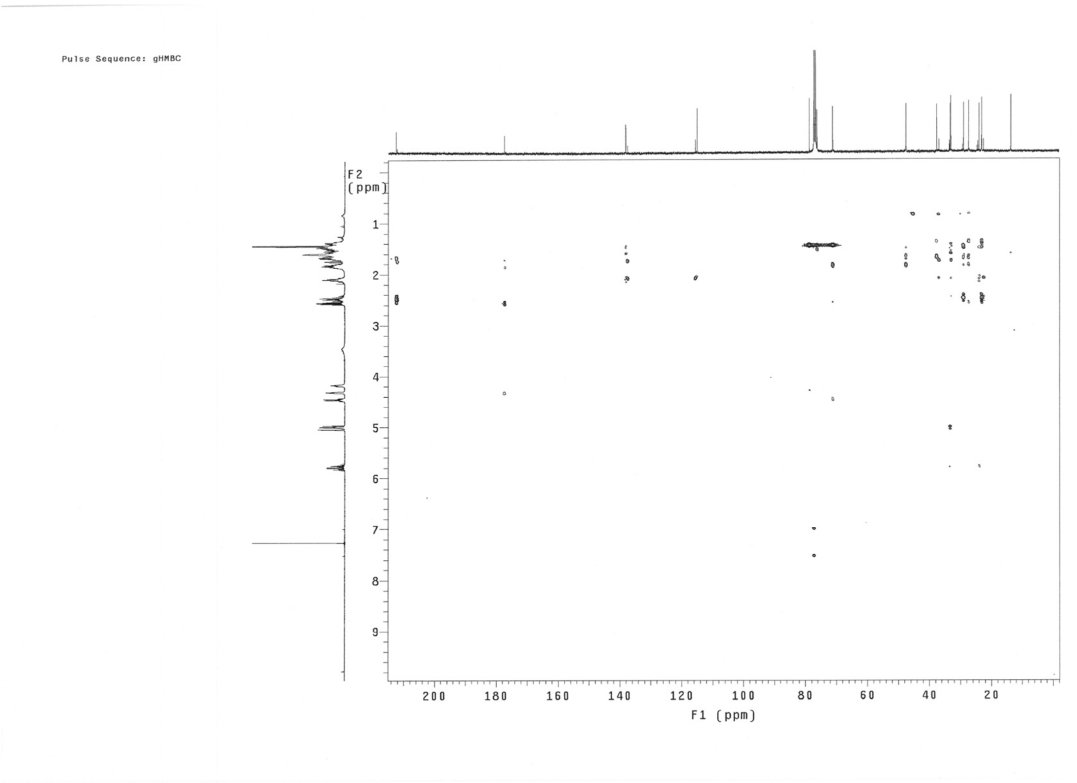

Figure S45. HMBC spectrum of **5**

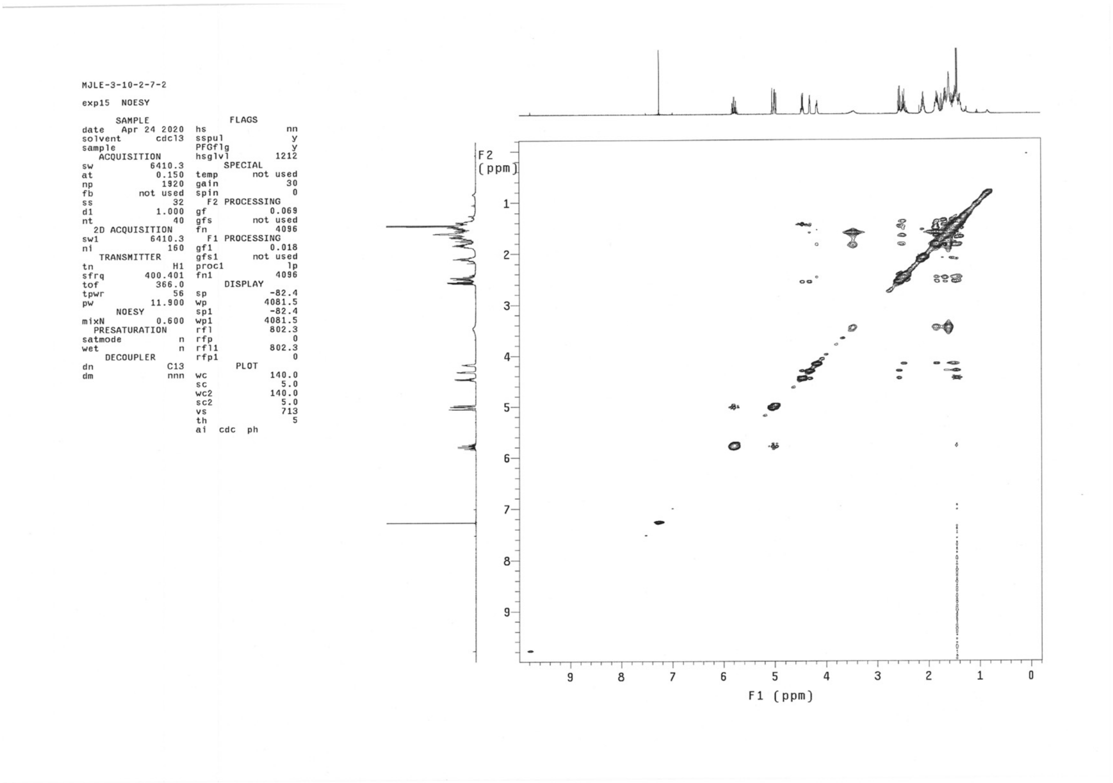

Figure S46. NOESY spectrum of **5**

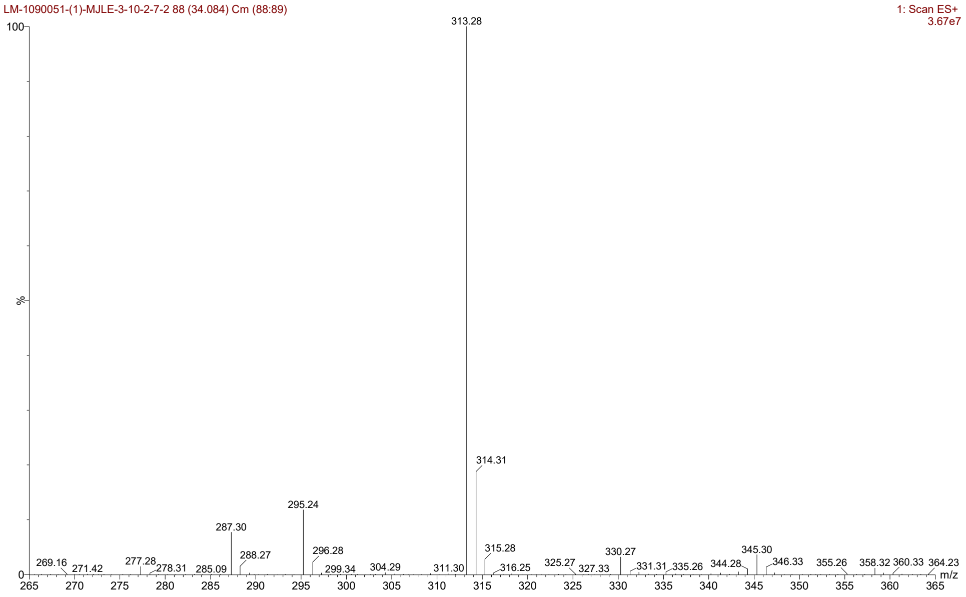

Figure S47. ESIMS spectrum of **5**

Mass Spectrum SmartFormula Report

Analysis Info

Analysis Name	D:\Data\f7\MJLE310272_000002.d	5/14/2020 4:18:37 PM
Method	broadband first signal	Operator: YU HSIAO-CHING
Sample Name	MJLE-3-10-2-7-2	Instrument: BRUKER FT-MS solariX
Comment	ESI Positive	

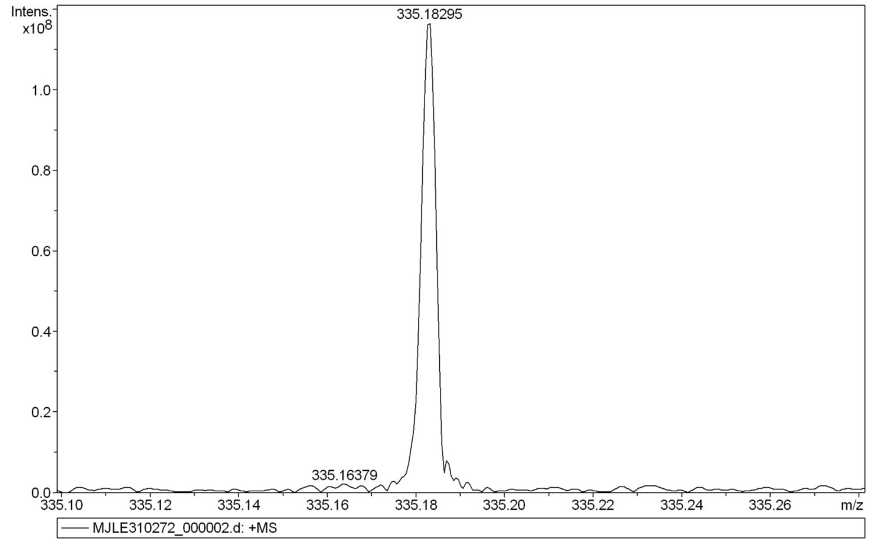

Meas. m/z	#	Formula	Score	m/z	err [mDa]	err [ppm]	mSigma	rdb	e⁻ Conf	N-Rule
335.18295	1	C 17 H 28 Na O 5	100.00	335.18290	-0.05	-0.16	17.2	3.5	even	ok

Figure S48. HRESIMS spectrum of **5**

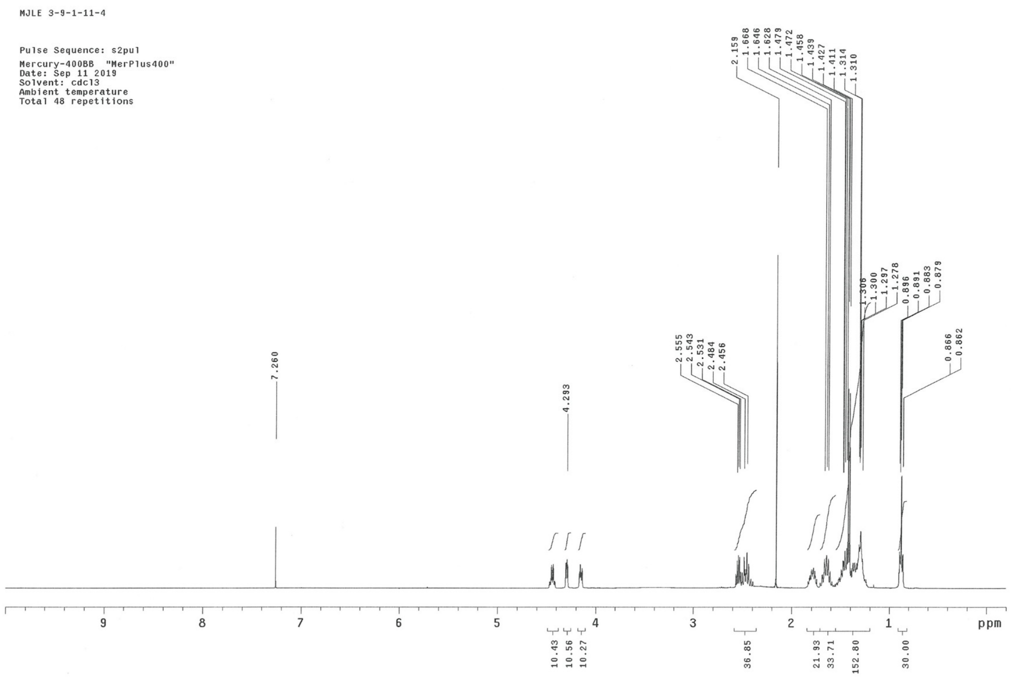

Figure S49. ^{1}H NMR spectrum of (400 MHz, $CDCl_3$) spectrum of **6**

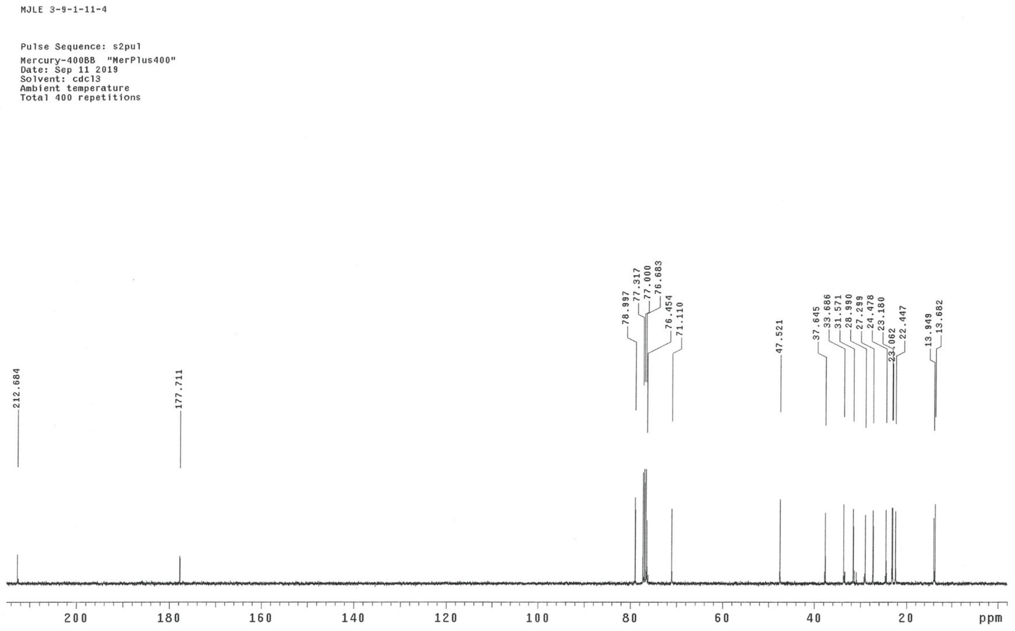

Figure S50. ^{13}C NMR spectrum of (100 MHz, $CDCl_3$) spectrum of **6**

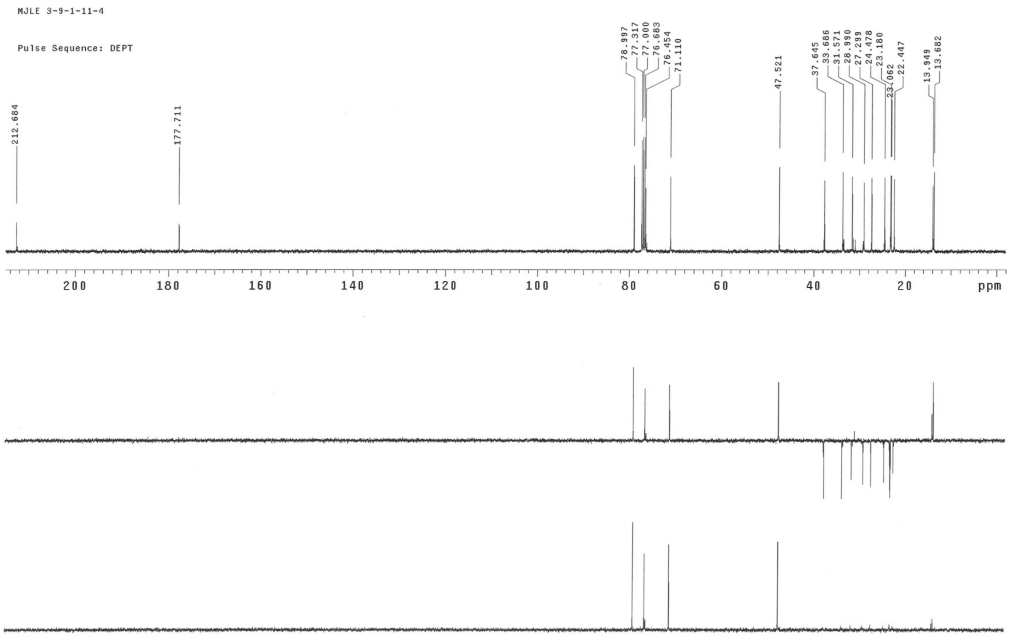

Figure S51. DEPT spectrum of **6**

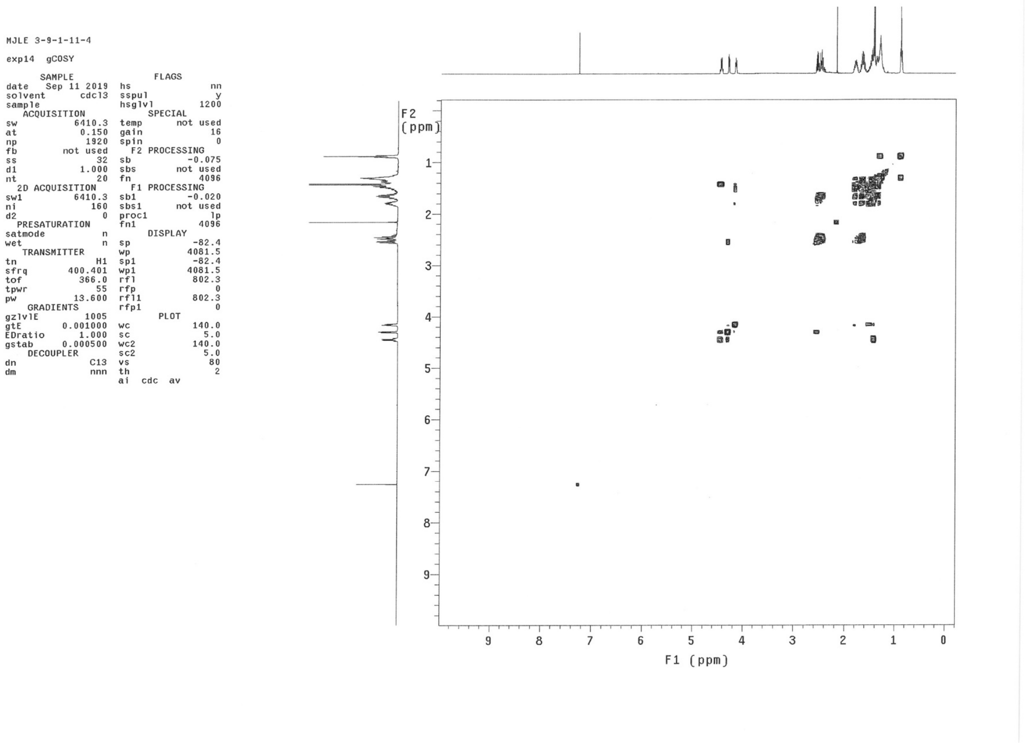

Figure S52. COSY spectrum of **6**

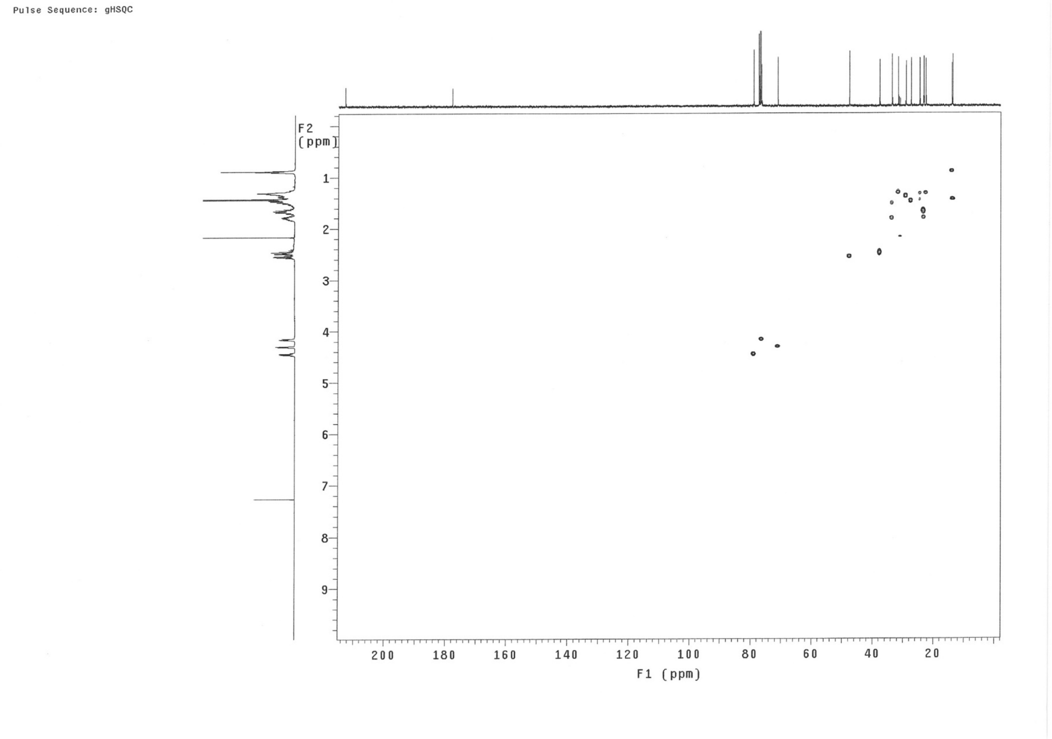

Figure S53. HSQC spectrum of **6**

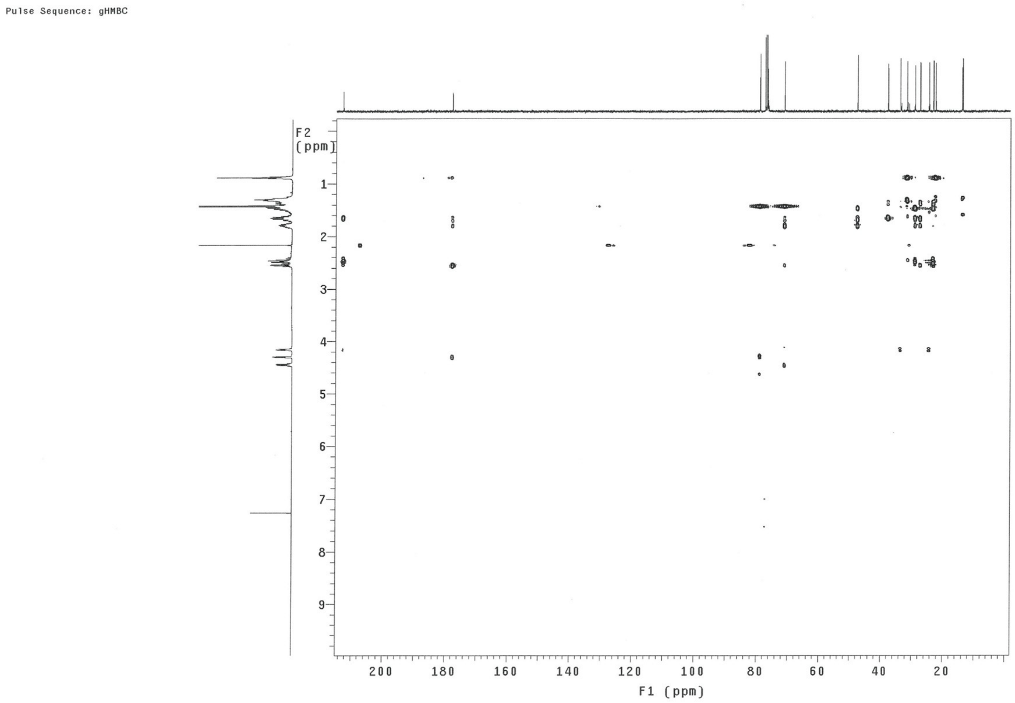

Figure S54. HMBC spectrum of **6**

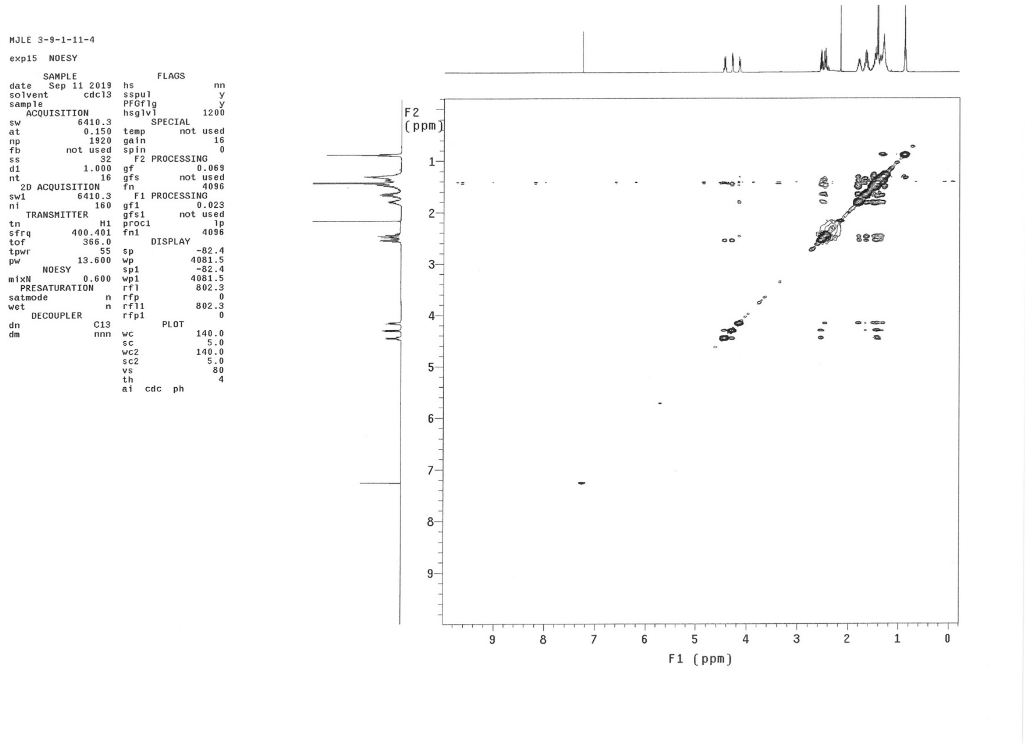

Figure S55. HMBC spectrum of **6**

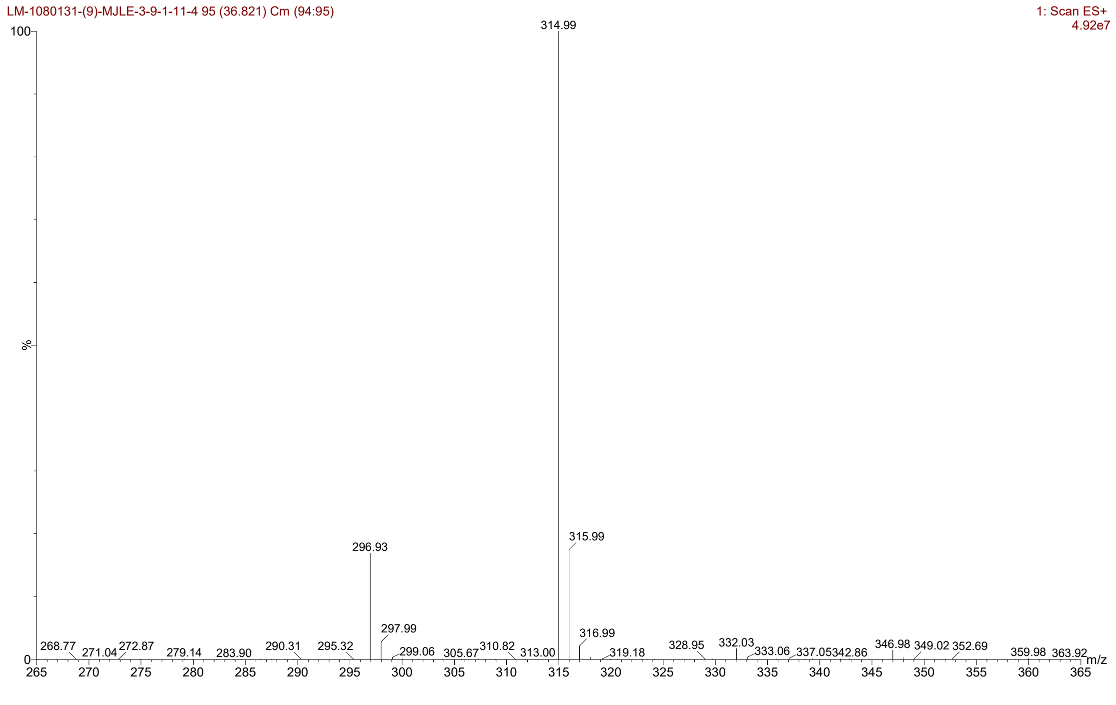

Figure S56. ESIMS spectrum of **6**

Mass Spectrum SmartFormula Report

Analysis Info

Analysis Name	D:\Data\f7\MJLE391114_000004.d	12/31/2019 2:44:51 PM
Method	broadband first signal	Operator: YU HSIAO-CHING
Sample Name	MJLE-3-9-1-11-4	Instrument: BRUKER FT-MS solariX
Comment	ESI Positive	

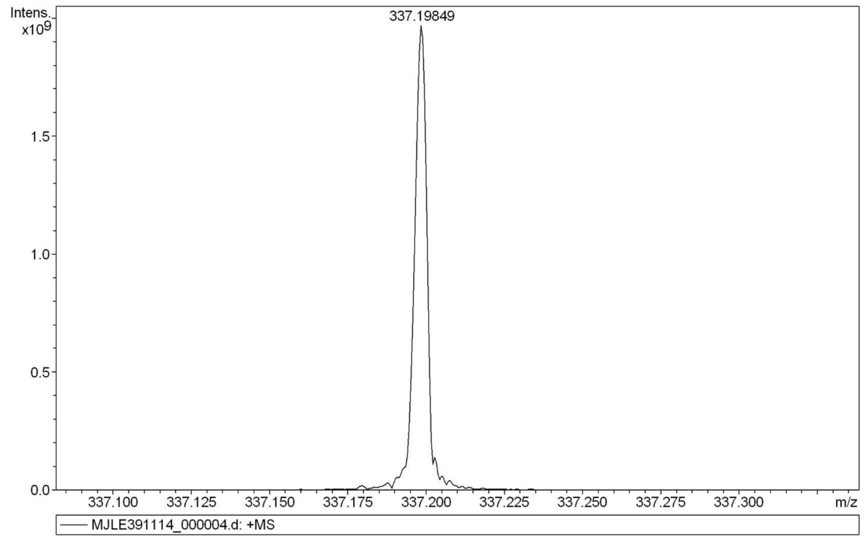

Meas. m/z	#	Formula	Score	m/z	err [mDa]	err [ppm]	mSigma	rdb	e⁻ Conf	N-Rule
337.19849	1	C 17 H 30 Na O 5	100.00	337.19855	0.05	0.16	15.9	2.5	even	ok

Figure S57. HRESIMS spectrum of **6**

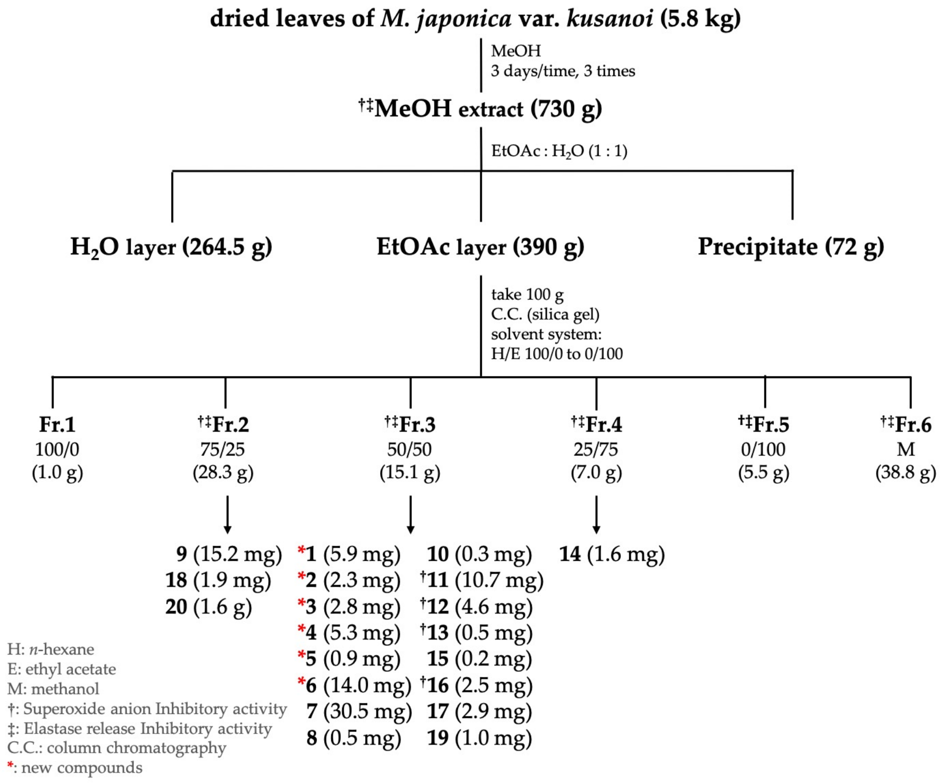

Figure S58. Extraction and isolation of the leaves from *M. japonica* var. *kusanoi*.

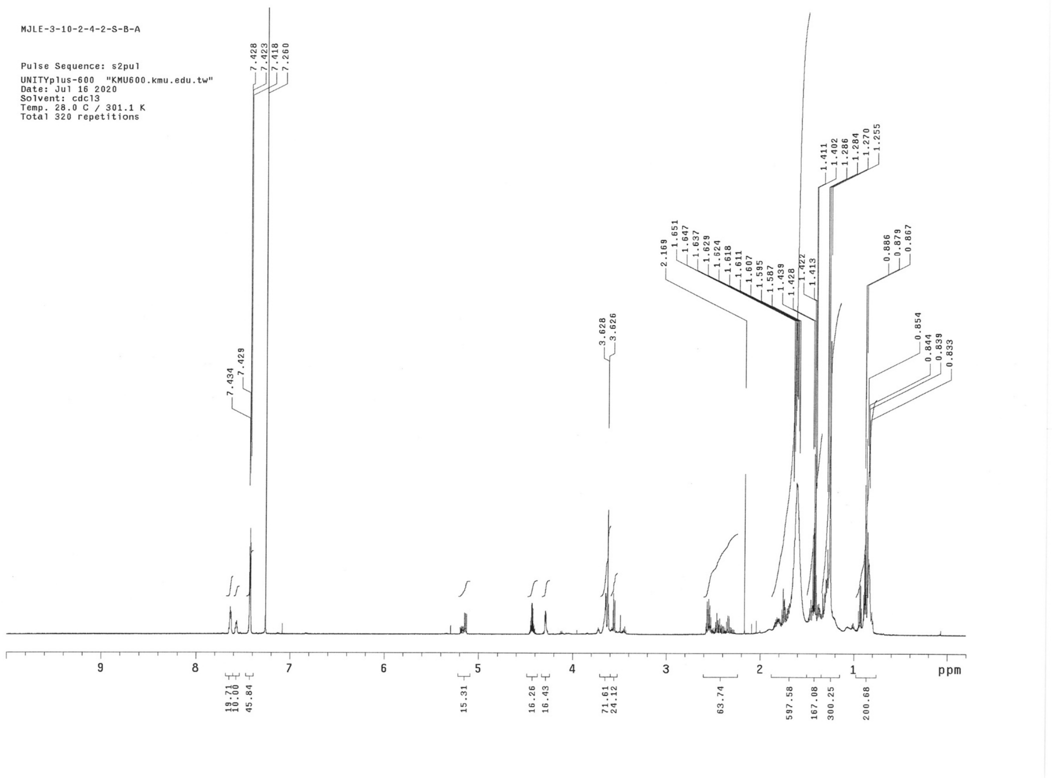

Figure S59. ^{1}H NMR spectrum of (600 MHz, $CDCl_3$) spectrum of **4a**

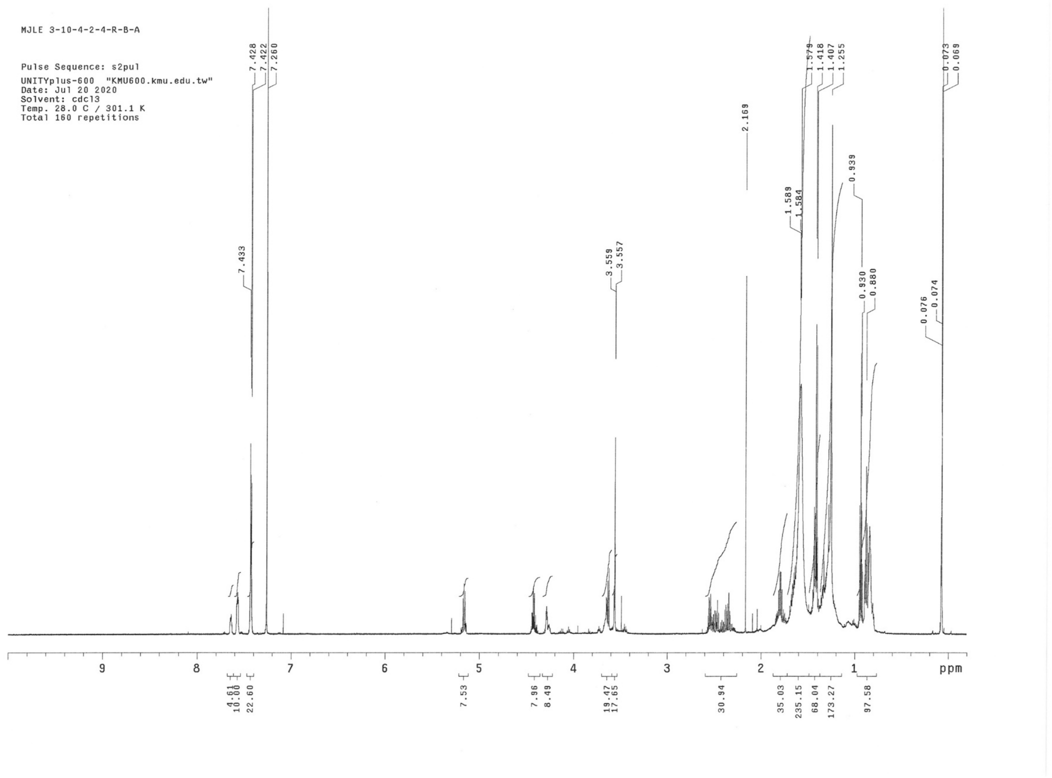

Figure S60. ^{1}H NMR spectrum of (600 MHz, $CDCl_3$) spectrum of **4b**

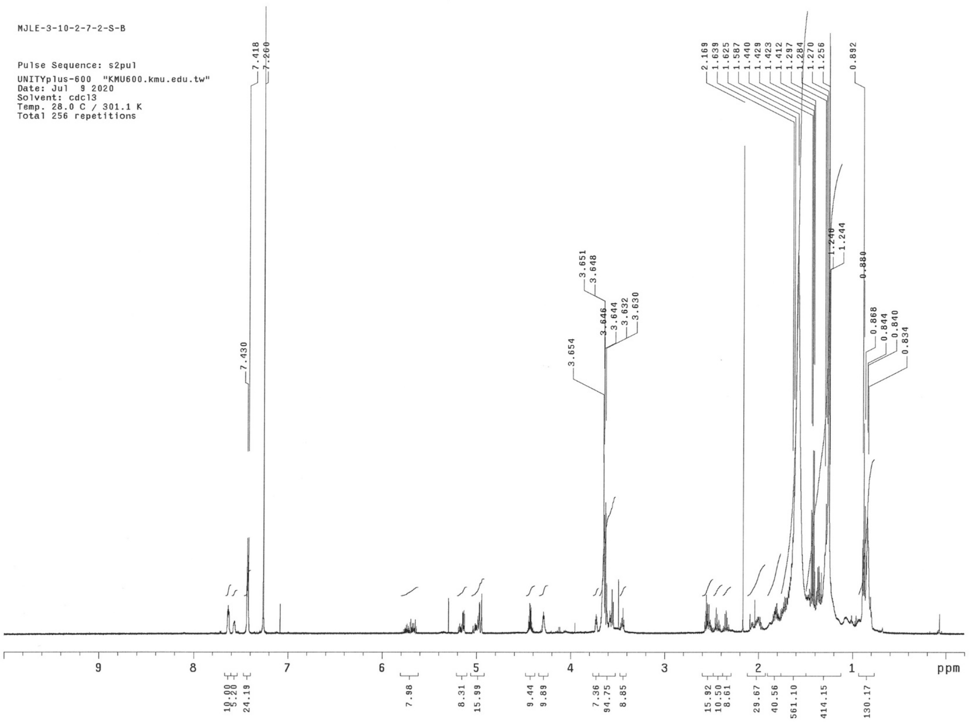

Figure S61. ¹H NMR spectrum of (600 MHz, $CDCl_3$) spectrum of **5a**

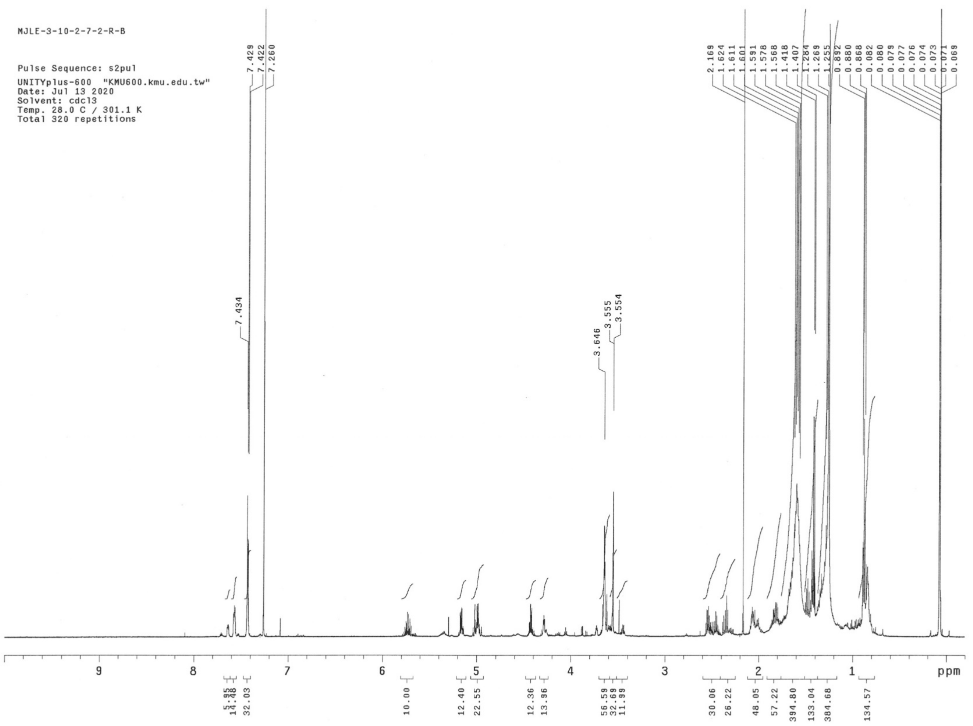

Figure S62. ¹H NMR spectrum of (600 MHz, $CDCl_3$) spectrum of **5b**

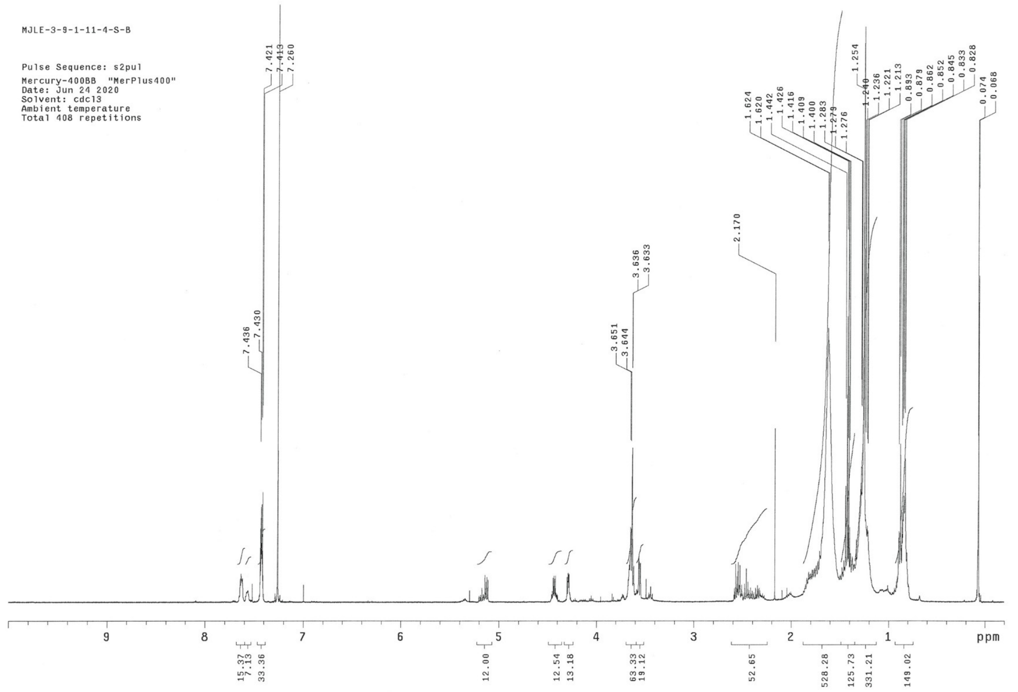

Figure S63. ^{1}H NMR spectrum of (400 MHz, $CDCl_3$) spectrum of **6a**

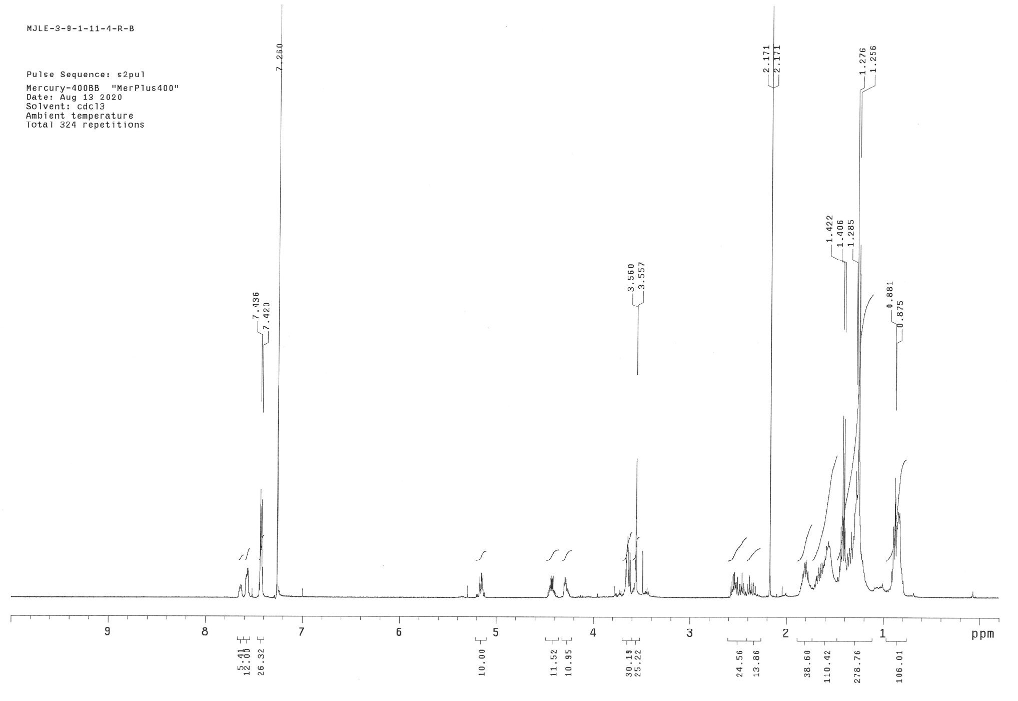

Figure S64. ^{1}H NMR spectrum of (400 MHz, $CDCl_3$) spectrum of **6b**

Table S1.Inhibitory effects of crude extracts from the leaves of *M. japonica* var. *kusanoi* on superoxide anion generation and elastase release in fMLP/CB-induced human neutrophils.

**Extract**	**Superoxide anion**	**Elastase release**
**(10 μg/mL)**	**Inh %**	**Inh %**
MJLW-1	36.25 ± 5.65	89.86 ± 4.77
MJLW-2	75.35 ± 5.12	95.00 ± 3.86
MJLW-3	71.13 ± 6.98	64.26 ± 2.58
MJLW-4	94.32 ± 0.89	128.43 ± 5.95
MJLW-5	108.63 ± 1.86	121.81 ± 4.24
MJLE-1	8.88 ± 7.27	17.92 ± 2.03
MJLE-2	103.05 ± 0.80	128.41 ± 0.78
MJLE-3	101.85 ± 1.92	120.57 ± 6.06
MJLE-4 （1 μg/mL）	102.45 ± 0.41	118.19 ± 5.59
MJLE-5	98.28 ± 2.92	117.61 ± 4.35
MJLE-6	110.84 ± 2.02	119.78 ± 5.15

Percentage of inhibition (Inh %) at 10 μg/ml concentration. MJLW: water layer from the leaves of *M. japonica* var. *kusanoi*, MJLE: ethyl acetate layer from the leaves of *M. japonica* var. *kusanoi*.
